# A nuclear orthologue of the dNTP triphosphohydrolase SAMHD1 controls dNTP homeostasis and genomic stability in *Trypanosoma brucei*

**DOI:** 10.3389/fcimb.2023.1241305

**Published:** 2023-08-22

**Authors:** Pablo Antequera-Parrilla, Víctor M. Castillo-Acosta, Cristina Bosch-Navarrete, Luis Miguel Ruiz-Pérez, Dolores González-Pacanowska

**Affiliations:** Instituto de Parasitología y Biomedicina “López-Neyra, Consejo Superior de Investigaciones Científicas, Parque Tecnológico de Ciencias de la Salud, Granada, Spain

**Keywords:** nucleotide, pyrimidine metabolism, *Trypanosoma brucei*, deoxynucleoside triphosphate homeostasis, genomic stability

## Abstract

Maintenance of dNTPs pools in *Trypanosoma brucei* is dependent on both biosynthetic and degradation pathways that together ensure correct cellular homeostasis throughout the cell cycle which is essential for the preservation of genomic stability. Both the salvage and *de novo* pathways participate in the provision of pyrimidine dNTPs while purine dNTPs are made available solely through salvage. In order to identify enzymes involved in degradation here we have characterized the role of a trypanosomal SAMHD1 orthologue denominated TbHD82. Our results show that TbHD82 is a nuclear enzyme in both procyclic and bloodstream forms of *T. brucei*. Knockout forms exhibit a hypermutator phenotype, cell cycle perturbations and an activation of the DNA repair response. Furthermore, dNTP quantification of *TbHD82* null mutant cells revealed perturbations in nucleotide metabolism with a substantial accumulation of dATP, dCTP and dTTP. We propose that this HD domain-containing protein present in kinetoplastids plays an essential role acting as a sentinel of genomic fidelity by modulating the unnecessary and detrimental accumulation of dNTPs.

## Introduction

The protozoan parasite *Trypanosoma brucei* is responsible for Human African Trypanosomiasis (HAT) or sleeping sickness and has been well-studied as a model system for the understanding of kinetoplastid biology (Romero-Meza and Mugnier, 2020). The establishment of unique features in this parasite could lead to the identification of novel drug targets and strategies for therapeutic intervention.

The maintenance of an adequate pool of the four canonical deoxyribonucleotides (dNTPs) is essential for the correct synthesis and repair of DNA. Both synthetic and degradation pathways operate in this process (Pai and Kearsey, 2017; Liu et al., 2022). In kinetoplastid parasites the salvage and *de novo* pathways participate in the provision of pyrimidine dNTPs while purine dNTPs are made available solely through salvage. Catabolic pathways involved in dNTP degradation involve enzymes such as 5’-nucleotidases, nucleoside phosphorylases, and deaminases. The regulation of the levels of dNTPs through catabolism throughout the cell cycle is a well-known process in mammalian cells while the information available in trypanosomes is limited. In our search for nucleotide metabolizing enzymes in the *T. brucei* genome, we have identified two HD domain-containing nucleotide hydrolases, TbHD52 and TbHD82 (Yague-Capilla et al., 2021), that are strongly related to human sterile alpha motif and histidine-aspartic acid domain-containing protein 1 (SAMHD1). Human SAMHD1 is a dNTP triphosphohydrolase that crucially controls the homeostatic balance of cellular dNTPs and also acts as an immunodeficiency virus (HIV)-1 restriction factor (Goldstone et al., 2011; Laguette et al., 2011; Powell et al., 2011; Kim et al., 2012; Lahouassa et al., 2012; Franzolin et al., 2013). In addition to its antiviral role, SAMHD1-mediated regulation is necessary for efficient DNA repair and colocalizes with DNA repair foci in cells exposed to genotoxic agents (Daddacha et al., 2017). It has also been shown that SAMHD1 promotes degradation of nascent DNA at stalled replication forks in human cell lines by stimulating the exonuclease activity of MRE11 (Coquel et al., 2018).

We have previously characterized the smaller version of the two trypanosomal SAMHD1 orthologues, TbHD52, which exhibits a 24.7% amino acid sequence identity with TbHD82. The enzyme is mitochondrial and null mutants are pyrimidine auxotrophs and present an extensive accumulation of dCTP and derived metabolites. Thus, we proposed that TbHD52 plays an essential role in the provision of mitochondrial intermediates important for cellular pyrimidine dNTP homeostasis (Yague-Capilla et al., 2021).

Here we report the characterization of the second *T. brucei* SAMHD1 orthologue, TbHD82. The aim of the present study was to clarify the participation of this protein in the control of dNTP levels in this parasite. To gain further insight into this putative NTP pyrophosphatase, we performed an analysis of TbHD82-deficient cells. The data presented suggest that TbHD82 has a primary role in the homeostasis of cellular dNTPs and while the absence of this role does not result in significant defects in proliferation, S phase progression is perturbed in *TbHD82* null mutants. Furthermore, TbHD82 presents a nuclear localization, expression is cell cycle-regulated and its function is important for the maintenance of genomic integrity. In summary, we propose that TbHD82 is involved in the control of dNTPs available for DNA replication and repair in the nucleus exerting an important role in dNTP homeostasis and the preservation of genomic integrity.

## Methods

### Trypanosome growth and transfection

The cell lines *Tb HD82*-dKO, *Tb HD82*-cdKO, *Tb* HD82-OE and *Tb* HD82-myc were generated for both the *T. brucei* single-marker bloodstream form (BF) (Wirtz et al., 1999) and the *T. brucei* 449 procyclic form (PF) (Estevez et al., 2001). Bloodstream trypanosomes were cultured at 37°C and 5% CO_2_ in HMI-9 with 10% (v/v) fetal bovine serum (FBS) and procyclic parasites were cultured at 28°C in SDM-79 supplemented with 10% FBS and 7.5 µg/ml hemin.

Parasites were transfected by electroporation in cytomix buffer for bloodstream cells and Zimmerman buffer for procyclic cells, as previously described (Castillo-Acosta et al., 2008). Clones were selected with appropriate selection drugs at the following concentrations: puromycin (Sigma): 0.1 µg/ml (BF) and 1 µg/ml (PF); hygromycin (Sigma): 5 µg/ml (BF) and 50 µg/ml (PF); phleomycin (Sigma): 2.5 µg/ml (BF) and blasticidin (Invitrogen): 5 µg/ml (BF) and 10 µg/ml (PF). Expression of the ectopic copy in TbHD82-OE and TbHD82-myc was induced using 1 µg/ml of doxycycline (DOX) (Sigma).

The TbHD82 alleles replacement (*Tb HD82*-dKO) was performed by transfection of two gene cassettes containing the blasticidin S deaminase (*BSD*) or hygromycin phosphotransferase (*HYG*) resistance markers.

Overexpression of both the native TbHD82 and a c-myc tagged fusion protein (TbHD82-myc) were achieved by transfection with pGRV190 and pGRV191 plasmids, respectively, linearized with NotI.

### Generation of the TbHD82 knockout cell line

Replacement of the two alleles of both BF and PF was attained by transfection with two gene cassettes containing the *BSD* or *HYG* resistance markers. These cassettes were generated by PCR amplification of a 5’-UTR fragment of 502 bp (position 330-831) and a 3’-UTR fragment of 340 bp (position 281-620) of the regions flanking the open reading frame of the TbHD82 gene using wild type *T. brucei* genomic DNA as template. Four specific primers were designed taking into account the sequence of Tb427.06.2900 present in the Gene DB database: 5’-CGC GGC CGC AAC TGA GAG GGT GAT TGG CG-3’ (NotI restriction site underlined) and 5’- GCC TCG AGT CGT GTG ATT TGC TAA CGC-3’ (XhoI) for the 5’-UTR region, and 5’-GCA GGC CTG TGC GCA TGT ACG TTG TAG C-3’ (StuI) and 5’-GGC TAG CGC GGC CGC CTA TCG TCC AAG TGT TTC TA-3’ (NheI and NotI) for the 3’-UTR region.

The 5’-UTR and 3’-UTR regions of the TbHD82 were introduced as flanking sequences of the *BSD* resistance gene in the pHD887 plasmid kindly provided by Christine Clayton (ZMBH, Heidelberg, Germany), yielding the construct pGRV188. The second cassette was generated by replacement of the *BSD* gene in the pGRV188 plasmid with the *HYG* selectable marker coming from SnaBI-digested pGR19 (Clayton et al., 2005), giving the construct pGRV189.

### Generation of the TbHD82-OE and TbHD82-myc cell lines

Studies on the intracellular localization of TbHD82 localization were performed in BF and PF cell lines that overexpress the TbHD82 native protein (*Tb* HD82-OE) or a TbHD82-c-myc fusion protein (*Tb* HD82-myc). The TbHD82 coding sequence (Tb427.06.2900) was amplified by PCR using wild type *T. brucei* genomic DNA as template and the specific primers: 5’-GCA TTA ATA TGG AAG GGG AAC TTG CCT TC-3’ (AseI restriction site underlined) and 5’-GCG GAT CCT TAG CGG CTG CGT TTA TTT CC-3’ (BamHI) for the native form and 5’-GCA TTA ATA TGG AAG GGG AAC TTG CCT TC-3’ (AseI) and 5’-GCG ATA TCG CGG CTG CGT TTA TTT CCC GC-3’(EcoRV) lacking a stop codon for expression of the c-myc-fusion protein. The fragment corresponding to the native TbHD82 was subcloned in pGRV23b (Castillo-Acosta et al., 2012b) after NdeI and BamHI digestion yielding pGRV190 plasmid. In the case of the c-myc fusion protein, the PCR fragment was subcloned in pGRV33 (Castillo-Acosta et al., 2012b) previously digested with NdeI and HpaI resulting in the pGRV191 plasmid containing the carboxy-terminal additional amino acid sequence SKGKVNEEQKLISEEDL* (c-myc sequence is underlined, the asterisk indicates a stop codon). Both the pGRV190 and pGRV191 plasmids allow for regulated expression by a tetracycline-inducible PARP promoter and puromycin (PAC resistance gene) was used as drug for selection.

### Western blot analysis

For western blot analysis, 5 x 10^6^ cells were harvested, centrifuged and washed in 1 × PBS (137 mM NaCl, 4 mM Na_2_HPO_4_, 1.7 mM KH_2_PO_4_ and 2.7 mM KCl). Pellets were resuspended in urea buffer (6 M urea, 10 mM Na_2_HPO_4_, 1% β-mercaptoethanol, pH 7) and loading sample buffer (67.5 mM Tris-HCl, pH 6.8, 3% SDS, 10% glycerol, 5% β-mercaptoethanol) in a 3:1 ratio, and boiled for 5 min at 99°C. Samples were subjected to electrophoresis in SDS-PAGE and transferred to polyvinylidene difluoride (PVDF) membranes, which were incubated with a rabbit polyclonal anti-TbHD82 (1:10,000) antibody generated against recombinant TbHD82 (Yague-Capilla et al., 2021). The mouse monoclonal anti-Tbβ-tubulin (1:10,000 [Sigma]) antibody was used as loading marker. Goat anti-rabbit IgG (1:5,000) or goat anti-mouse IgG (1:3,000) HPRT-conjugated antibodies (Promega) were used as secondary antibodies and films were developed using the ECL enhanced chemiluminescence detection kit (Amersham Pharmacia Biotech).

### Immunofluorescence studies

Immunofluorescence (IF) studies were performed for intracellular localization as well as the quantification of γH2A phosphorylation. Colocalization analysis was performed in BF and PF cell lines. Briefly, 5 x 10^6^ parasites were harvested and incubated in 1 ml of culture medium without FBS supplemented with 10 µM (BF) and 100 µM (PF) MitoTrackerVR Red CMXRos for 15 min at 37°C (BF) or 28°C (PF), followed by incubation in fresh culture medium without FBS and washing twice with 1 ml of PBS. In the case of γH2A phosphorylation IF, cells were directly washed and then the parasites were fixed in 4% *p*-formaldehyde (PFA) in wash solution (PBS, 0.2% Tween 20) on a poly-l-lysine coated slide for 20 min. Fixed parasites were washed once in wash solution and permeabilized and blocked with 1% of IGEPAL in blocking solution (wash solution containing 1% blocking reagent [Roche]) during 75 min. Samples were incubated with the corresponding primary antibody; anti-TbHD82 polyclonal antibody (1:100 diluted in blocking solution), monoclonal anti-c-myc (Sigma, 1:100 diluted in blocking solution), or the anti-TbγH2A polyclonal antibody (Life Technologies, 1:100 diluted in blocking solution). Samples were washed six times for 5 min and then labeled with secondary antibodies for 1 h at room temperature: goat Alexa Fluor 488 anti-rabbit (Sigma, 1:40 diluted in blocking solution) or goat Alexa Fluor 488 anti-mouse (Sigma, 1:40 diluted in blocking solution). Preparations were dehydrated in methanol for 1 min, mounted and stained with ProLong Gold Antifade Reagent with DAPI (Life Technologies). Vertical stacks of up to 40 slices (0.2 µm steps) were captured using an inverted Leica DMi8 microscope, 100x objective, and LASX software. Images were deconvolved and pseudo-colored with Huygens Essential software (version 3.3; Scientific Volume Imaging). Colocalization analysis was carried out using the JACoP plugin of the Fiji software (version 1.53q; ImageJ). For 3D images and videos, vertical stacks were captured using a sp8 confocal spectral Leica microscope, 63x objective, with hyvolution technology. Images were processed, rendered and clipped with LASX software (version 3.5.6).

### Measurement of intracellular nucleotides

The four canonical dNTPs (dTTP, dCTP, dATP and dGTP) were quantified in *Tb* BF, *Tb* BF *HD82*-dKO and *Tb* BF *HD82*-conditional knockout (*HD82*-cdKO; see **Figure S3**) cells in the absence or presence of DOX using a modified DNA polymerase assay (Horowitz et al., 1997). The oligonucleotides 5’-TTT ATT TAT TTA TTT ATT TAG GCG GTG GAG GCG G-3’, 5’-AAA CAA ACA AAC AAA CAA ACG GCG GAG GAG GCG G-3’, 5’-AAA GAA AGA AAG AAA GAA AGG GCG GAG GAG GCG G-3’, 5′-AAA TAA ATA AAT AAA TAA ATG GCG GTG GAG GCG G-3′ were employed as template for dTTP, dGTP, dCTP and dATP measurements respectively, and the oligonucleotide 5’-CCGCCTCCACCGCC-3’ as primer. Parasites (2 x 10^6^ cells per sample) were harvested by centrifugation and washed once in PBS. Cells were incubated with 250 µl of cold 60% methanol in PBS overnight at -20°C. The suspension was heated for 5 min in boiling water bath, followed by centrifugation for 20 min at 17,000 × *g*. The supernatant was moved to a fresh tube and dried in a Speed-Vac (Thermo Fischer Scientific). Residues obtained this way were then dissolved in 100 µL of reaction mixture containing 32 nM DNA template, 32 nM DNA primer, NEBuffer 2 (New England BioLabs), 0.3 units of DNA polymerase I Klenow fragment (New England Biolabs) and 0.0032 µCi/µl of the corresponding [^3^H]deoxynucleotide ([^3^H]dATP (PerkinElmer) for dTTP measurements and [^3^H]dTTP (Moravek) for dCTP, dATP and dGTP measurements). Samples were incubated for 15 min at 25°C. The polymerization was stopped by adding 10 mM EDTA and heating at 75°C for 20 min. DNA was then precipitated with 10% (v/v) trichloroacetic acid for 30 min at 4°C. The solution was filtered with Glass Microfibre Filters GF/C (Whatman) and the filters were washed under a vacuum with 30 ml of 5% trichloroacetic acid, rinsed with 3 ml of ethanol and dried. Radioactivity was measured in a LS 6500 Multi-Purpose Scintillation counter (Beckman Coulter). Data were interpolated in standard curves obtained using different concentrations of the corresponding dNTP.

### Fluorescence activated cell sorting (FACS) for cell cycle analysis

For FACS analysis, 1.5 x 10^6^ parasites were harvested by centrifugation, washed twice with TDB-g buffer (5 mM KCl, 80 mM NaCl, 1 mM MgSO_4_, 20 mM Na_2_HPO_4_, 20 mM glucose, pH 7.4) and permeabilized with 50 µl saponin diluted in TDB-g (0.05% saponin for BF and 0.1% saponin for PF parasites). Cells were incubated at 37°C (BF) and 28°C (PF) for 3 min and the reaction was stopped by adding 450 µl of 10 µg/ml RNase A diluted in TDB-g. Samples were incubated at room temperature for 30 minutes and then propidium iodide was added to a final concentration of 20 µg/ml. The content of the tubes was transferred to special cytometer tubes and analyzed in a FACSymphony A3 SE Cell Analyzer using BDFacsDiva version 8 (Becton Dickinson). Percentages of each phase of the cell cycle were analyzed with FlowJo version 10.7.1.

### Mutation rate and spectra

The rate of reversion to the *TK^-^* phenotype in PF cells transfected with the viral TK gene was measured by the Luria-Delbrück fluctuation test. Stock cultures were prepared at a density of 10^3^ cells/ml (log phase). The cultures were split in 96-well plates by adding 200 parasites per well. Cultures were incubated for 6 days until the density reached 5-10 x 10^6^ parasites/ml, then cultures were transferred to 24-well plates with 2.5 ml containing 100 µM of ganciclovir (Sigma) and the plates were sealed. Cultures were left growing during 8 days and then cell density was determined. The mutation rate was calculated using the equation described by Valdes et al. (1996) :


$$\alpha =\frac{\ln P_{0}\;\times \;\ln2}{N}$$

where P_0_ is the proportion of wells without cell growth and N is the final number of parasites per culture upon addition of ganciclovir.

In order to establish the mutation spectra, genomic DNA of independent *TK^-^* revertants was isolated using DNAzol (Invitrogen) and the *TK* gene amplified by PCR. The oligonucleotides VCA19 (5’-GAG CCG ATG CTT TTG ACA TGT TAG-3’) and VCA20 (5’-CTT GTG CGC TGT ACG TAA ATG TGT TGC-3’) were used to sequence the *TK* gene of the different revertant clones.

### Detection of DNA strand breaks by TUNEL

Levels of DNA fragmentation were measured by TUNEL (terminal deoxynucleotidyl transferase-mediated dUTP nick end labeling) using the *In situ* Cell Death Detection Kit, Fluorescein (Roche). For each sample, 1.5 x 10^7^ parasites were harvested by centrifugation and washed twice with ice cold PBS. Cells were fixed by incubating in 2% *p*-formaldehyde for 45 min at room temperature, washed twice with PBS, and permeabilized with 0.1% Triton X-100 in 0.1% sodium citrate for 2 min at 4°C. Samples were then stained with the TUNEL reaction mix (with fluorescein-dUTP) at 37°C for 1 h. FL1-H fluorescence was measured on 10,000 events in a FACSymphony A3 SE Cell Analyzer (Becton Dickinson). Percentages of TUNEL positive cells were analyzed with BDFacsDiva version 8 (Becton Dickinson).

### Protein alignment

Alignment of the amino acid sequences was performed with the online tool ClustalOmega (EMBL’s European Bioinformatics Institute). The results were transferred to JalView version 2.11 and coincident amino acids were colorized applying Clustal style.

### Statistical analysis

Data were represented as the mean ± SD for each group. SPSS Statistics and GraphPad Software were used for the comparison of data sets. The student’s t test was used in the analysis when two sets of data were compared. Either the two-way ANOVA or the Dunnet’s *post hoc* tests were used to calculate variances of data sets with normal distribution. Kolmogorov−Smirnov and Shapiro−Wilk tests were applied to verify data normality, and the equality of variances was analyzed with the Levene’s test. A *p* value ≤ 0.05 was considered statistically significant. To identify differences between populations with categorical data with n < 300 cells, a Fisher’s exact test was applied, whilst a Chi-square test was used when n > 300.

## Results

### TbHD82 is located in the nucleus and exhibits cell-cycle-regulated expression

We previously identified Tb927.6.2900 as a member of the HD-domain/phosphodiesterase (PDEase)-like superfamily of proteins which has a total of 13 members in *T. brucei* (5 members belong to the HD family, and 8 are classified as PDEases) and designated it as TbHD82 taking into account its molecular mass (Yague-Capilla et al., 2021). PANTHER (Protein ANalysis Through Evolutionary Relationships; http://www.pantherdb.org) suggests that Tb927.6.2900 is a deoxynucleoside triphosphate triphosphohydrolase. TbHD82 has a theoretical Mw of 81,734 Da consisting of 735 amino acids with a calculated pI of 7.53 encoded by a single-copy gene located on chromosome 6. According to the Alphafold2 prediction (Jumper et al., 2021; Varadi et al., 2022), TbHD82 secondary structure, showed in **Figure S1**, contains two disordered regions (amino acids 641-675 in purple and 713-735 in yellow) with no predicted function and a central region (amino acids 216-366 in red) containing the HD domain conserved within the protein superfamily.

Immunofluorescence analysis was performed in different cell lines in order to identify the intracellular localization of TbHD82. For this purpose, we generated BF and PF cell lines with a tetracycline-controlled inducible expression system that allowed for overexpression of the protein, named as *Tb* BF HD82-OE and *Tb* PF HD82-OE. We generated an affinity-purified rabbit polyclonal anti-TbHD82 antibody which was used for determination of enzyme levels upon induction (Yague-Capilla et al., 2021). This antibody specifically recognizes TbHD82 in western blots of parasite extracts (**Figure S2E**). Additionally, we transfected BF and PF cells with a construct encoding a TbHD82-myc fusion protein in order to further evaluate the localization of the protein by using a monoclonal antibody directed against the c-myc epitope.

Immunoblots of *T. brucei* cell lysates after 2 days of induction showed that anti-TbHD82 recognized the wild-type (WT) TbHD82 protein as well as the endogenous C-terminally myc-tagged version of TbHD82 in both BF and PF (**Figure S2**).

**Figure 1:**
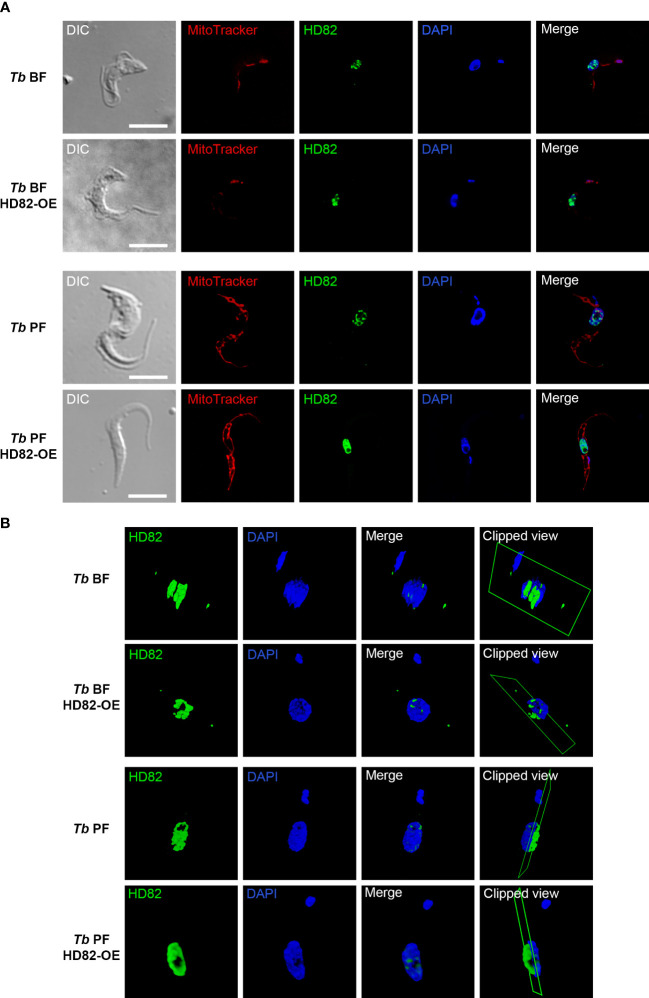
Immunofluorescence images of the intracellular localization of TbHD82 in both BF and PF parental as well as in overexpressing cell lines (*Tb* BF HD82-OE, *Tb* PF HD82-OE). **(A)** TbHD82 signal (green) was detected by using an affinity-purified polyclonal anti-TbHD82 as primary antibody and Alexa Fluor 488 conjugated anti-rabbit as secondary antibody. Images were captured using a Leica DMi8 microscope and LASX software (version 3.5.6). **(B)** 3D images obtained from a sp8 confocal spectral Leica microscope and LASX software (version 3.5.6). Nuclear and kinetoplast DNA were stained with DAPI, while the mitochondrion was stained with MitoTracker Red CMXRos. Bar, 5 μm.

The anti-TbHD82 antibody labeling revealed a nuclear localization in both BF and PF parental parasites (**Figure 1A**, lines 1 and 3). Cell lines which overexpress the untagged protein, *Tb* BF HD82-OE and *Tb* PF HD82-OE (**Figure 1A**, lines 2 and 4) also exhibit colocalization with nuclear DAPI staining. Mitotracker red staining was additionally performed to discard presence of the protein in the mitochondrion. **Figure 1B** and **Supplementary Videos** show the 3D rendering of nucleus and kinetoplast of parasites from the cell lines described above, extracted from the confocal stack.

A colocalization analysis with the Alexa Fluor 488 and DAPI signals was performed for n = 10 cells in each cell line. Pearson’s coefficients of 0.72 ± 0.06, 0.72 ± 0.07, 0.61 ± 0.08 and 0.73 ± 0.05 were obtained for the *Tb* BF, *Tb* BF HD82-OE, *Tb* PF and *Tb* PF HD82-OE and cell lines, respectively, hence supporting the nuclear localization.

**Figure 2:**
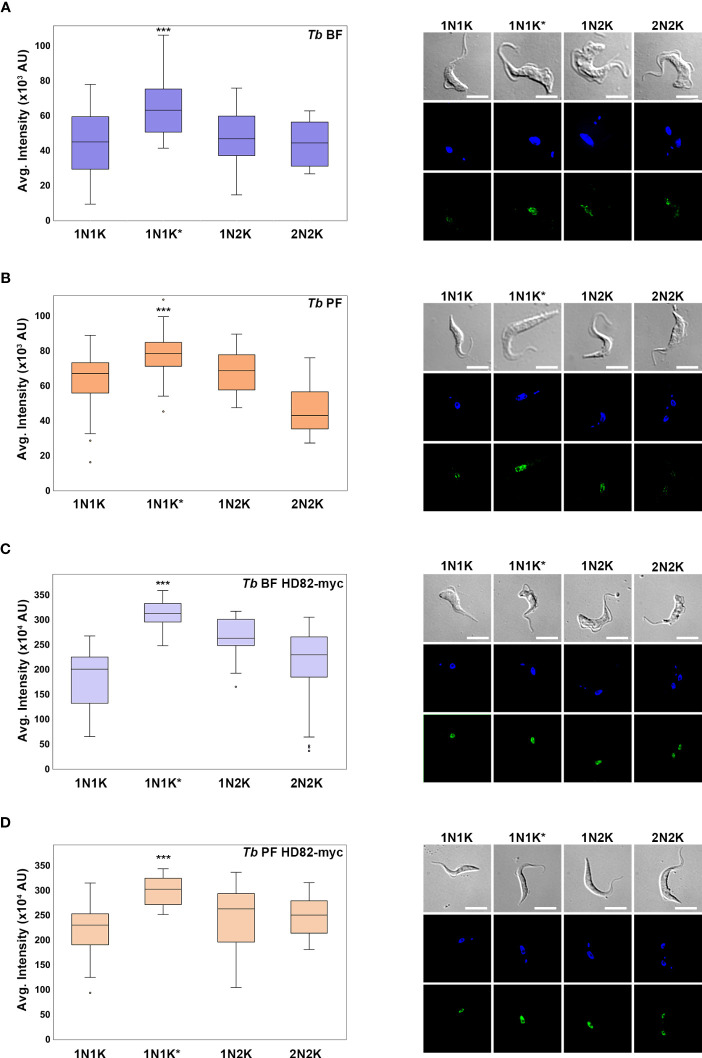
Analysis of the expression of TbHD82 during the cell cycle in *T. brucei*. TbHD82 expression levels were monitored by immunofluorescence analysis using an anti-TbHD82 and anti-c-myc as primary antibodies and Alexa Fluor 488 conjugated anti-rabbit and anti-mouse as secondary antibody, respectively. Images were collected with a Leica DMi8 and LASX software (version 3.5.6), followed by a deconvolution and pseudo-coloring using Huygens Essential software (version 3.3; Scientific Volume Imaging) and Fiji software (version 1.53q; ImageJ), respectively. Nuclei (N) and kinetoplast **(**K) were detected by DAPI staining and the differential interference contrast (DIC) image is shown as a grayscale. Cells were categorized according to the number of N and K in 1N1K, 1N1K*, 1N2K and 2N2K populations, where asterisks denote an enlarged K under segregation. Right panels illustrate an example of the HD82 signal for each cell cycle population and each cell line. Levels of TbHD82 were analysed in BF and PF parental parasites using an anti-TbHD82 antibody **(A, B)** as well as in cells overexpressing a c-myc tagged fusion protein, *Tb* BF HD82-myc and *Tb* PF HD82-myc, using an anti-c-myc antibody **(C, D)**. The signal intensity of HD82 was plotted in boxes graphs, which represent the values between the 25^th^ and 75^th^ percentile for each population, while whiskers represent minimum and maximum values. Dots represent atypical values. A one-way ANOVA test was carried out to check the presence of statistical differences between populations. The Student’s t test was used to identify significant differences. ***p < 0.001.

It is known that certain proteins involved in dNTP homeostasis are regulated throughout the cell cycle (Chang et al., 1998; Valente et al., 2016). In the case of human SAMHD1, its function is regulated mainly by two factors: phosphorylation/dephosphorylation and changes in expression (Cribier et al., 2013; Schott et al., 2018). We sought to analyze if expression of TbHD82 is also regulated. For this purpose, levels of TbHD82 in more than 200 cells in both BF and PF of parental and overexpressing the TbHD82-myc fusion protein were measured by immunofluorescence. Cells were classified according to the number of nuclei and kinetoplasts (1N1K, 1N1K*, 1N2K and 2N2K). The quantification of the signal was performed with Fiji software (version 1.53q; ImageJ) (Schindelin et al., 2012), evaluating the intensity of the nucleus of the parasites. In all cases TbHD82 is present throughout the cell cycle yet increased expression was evidenced in 1N1K* cells, which correspond mainly to early S phase cells (Woodward and Gull, 1990) (**Figure 2**). Colocalization analysis with the Alexa Fluor 488 and DAPI signals was also performed for parasites expressing the c-myc fusion protein. Pearson’s coefficients obtained were 0.71 ± 0.03 and 0.76 ± 0.04 for *Tb* BF HD82-myc and *Tb* PF HD82-myc, respectively.

### Levels of dATP, dCTP and dTTP are modified in *TbHD82* null mutants

**Figure 3:**
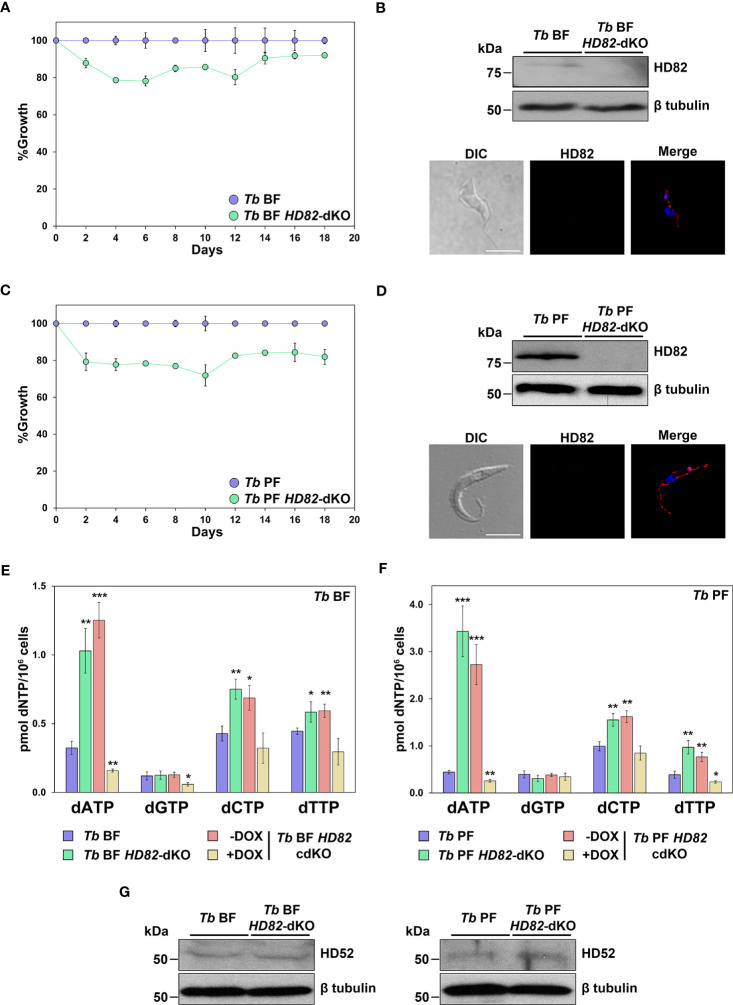
Analysis of parasite proliferation and dNTP profiling. **(A, C)** Plots showing the growth percentages in both BF **(A)** and PF **(C)** of HD82-deficient parasites compared to parental lines. Proliferation was measured in three independent biological replicates. **(B, D)** western blotting and immunofluorescence analysis were carried out to check the absence of HD82 in the null mutants using the anti-TbHD82 antibody and HPRT-conjugated anti-rabbit as secondary antibody. HD82 signal in western blotting was normalized using an anti-Tbβ-tubulin (Tbβ-tubulin, 50 kDa) as loading control. For immunofluorescence, nuclear and kinetoplast DNA were stained with DAPI (blue), while the mitochondrion was stained with MitoTracker Red CMXRos (red). Bar, 5 μm. **(E, F)** dNTPs levels were evaluated by polymerase assay-based method in parental (*Tb* BF and *Tb* PF), null mutants (*Tb* BF *HD82*-dKO and *Tb* PF *HD82*-dKO) as well as in HD82-conditional knockout (*Tb* BF *HD82*-cdKO and *Tb* PF *HD82*-cdKO) ± DOX. **(G)** TbHD52 levels were checked in HD82-deficient cells using an anti-TbHD52 antibody (Yague-Capilla et al., 2021) and HD52 signal was normalized using an anti-Tbβ-tubulin (Tbβ-tubulin, 50 kDa) as loading control. Data are presented as mean concentration and standard deviation (± SD) corresponding to three independent biological replicates. The asterisks show significant differences *vs* the parental line, calculated by the Student’s t test. *p < 0.05, **p < 0.01, ***p < 0.001.

In order to decipher the role of TbHD82 in proliferation and nucleotide homeostasis in *T. brucei*, we generated *TbHD82* knockout mutant (*Tb HD82*-dKO) cell lines in both BF and PF parasites. *TbHD82* null mutants were obtained through the replacement of two alleles with cassettes containing the *BSD* or *HYG* resistance markers (**Figure S3A**). Transfectants were verified by PCR, using primers of the selectable markers and flanking genes upstream and downstream the *HD82* gene in addition to specific primers of the *HD82* gene open reading frame, which confirmed the absence of both alleles coding for TbHD82 (**Figures S3B, C**). The determination of protein levels in knockout cells was performed by western blotting using the anti-TbHD82 rabbit polyclonal antibody (**Figures 3B, D**). Immunoblots of *T. brucei* cell lysates showed that the anti-TbHD82 antibody recognized the wild-type (WT) protein (81.73 kDa) in BF and PF cells, while no staining was detected in knockout cells. *Tb HD82*-dKO cells do not exhibit modifications in the levels of TbHD52 thus discarding participation of the mitochondrial orthologue in possible compensatory mechanisms involved in modulation of dNTP pools in null mutants (**Figure 3G**).

In order to evaluate parasite proliferation, cultures were initiated at 3 x 10^3^ (BF) or 2 x 10^5^ (PF) parasites/ml and monitored during 18 days by counting in a Z1 Coulter counter. Cultures were diluted every 2 days to the initial density and maintained in the absence of the antibiotics previously used to generate the KO cell lines. Both the BF and PF *HD82* null mutant cell lines showed a cell density that was slightly reduced compared to that of the parental strains (**Figures 3A, C**).

To validate observations obtained with the knockout lines, we generated a conditional knockout cell line containing a doxycycline-inducible ectopic copy of the *HD82* gene (*HD82*-cdKO) with puromycin (*PAC*) as a resistance marker for both BF and PF (**Figure S3A**). The conditional knockout allows for doxycycline-mediated control of the expression of HD82 in the null mutant. While the increased levels of protein observed upon induction of the ectopic copy (5-fold and 6-fold in *Tb* BF *HD82*-cdKO and *Tb* PF *HD82*-cdKO cells, respectively) do not result in pronounced cytotoxicity (**Figure S4**), the growth defects observed in the *HD82*-dKO cell line were not efficiently reverted upon overexpression. Thus, conditional knockout cell lines ± DOX exhibit growth profiles comparable to *HD82*-dKO cells.

Since members of the deoxynucleoside triphosphate triphosphohydrolase family have a role in maintaining dNTP homeostasis by hydrolyzing all four canonical dNTPs (Franzolin et al., 2013), we aimed to elucidate whether HD82 shares this function with its human homologue. For this purpose, we performed polymerase-based dNTP quantification as previously described (Castillo-Acosta et al., 2008; Requena et al., 2014). In the absence of TbHD82, a significant expansion of the dATP, dCTP and dTTP pools was observed while the levels of dGTP remained unchanged. These alterations in null mutants were observed in a similar fashion in both BFs and PFs and were mostly reverted when the expression of the ectopic copy of the gene was induced (**Figures 3E, F**). Thus, dATP, dCTP and dTTP were increased 3.2, 1.8 and 1.3 -fold, respectively, in BF null mutants and 7.8, 1.6, and 2.5-fold, respectively, in *Tb* PF *HD82*-dKO cells. Furthermore, DOX-induced expression of an ectopic copy of TbHD82 resulted in certain instances in lower dNTP levels (**Figures 3E, F**), and this dysregulation might be responsible for the defects in proliferation observed. Hence, the data suggests that TbHD82 is a major player in controlling dATP, dCTP and dTTP levels.

### Lack of TbHD82 leads to defects in cell cycle progression, increased DNA damage and a hypermutator phenotype

**Figure 4:**
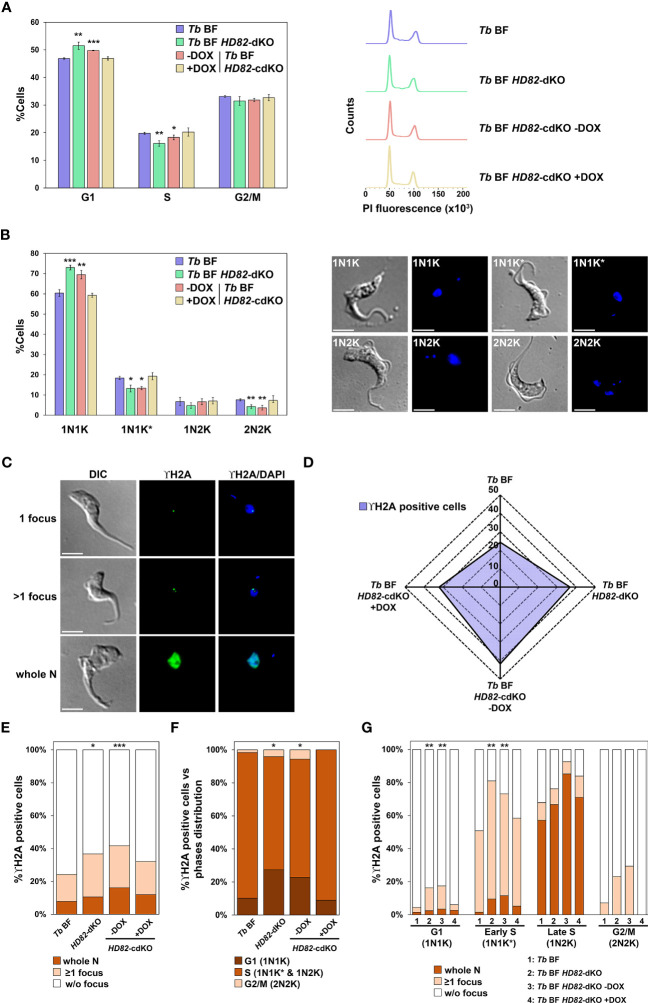
HD82-deficient BF cells exhibit cell cycle defects and an activation of the DNA damage response. For this purpose, *Tb* BF, *Tb* BF *HD82*-dKO as well as *Tb* BF *HD82*-cdKO ± DOX cell lines were analyzed. **(A)** Graph (left panel) indicates the percentages of cells in each of the different cell cycle stages determined by FACS analysis: G1, S and G2/M. Right panel illustrates an example of the histograms obtained for each cell line from the analysis. **(B)** Nuclei (N) and kinetoplasts (K) were stained with DAPI and cells were categorized according to the number of N and K in 1N1K, 1N1K*, 1N2K and 2N2K populations (asterisk denotes an enlarged K under segregation). The left panel shows quantification of the different populations and the right panel contains images illustrating each population. Data are presented as the mean percentage (± SD) of total cells counted from three independent experiments (n > 150 cells in total). **(C)** Immunofluorescence images were obtained using an anti-TbγH2A primary antibody and an Alexa Fluor 488-conjugated anti-rabbit secondary antibody. Positive cells are considered when at least one nuclear γH2A focus or whole nucleus staining was observed. **(D)** Radial plot showing the percentage of γH2A positive cells (purple area) determined by immunofluorescence microscopy (n > 150 for each sample from three independent replicates) as described in **(C)**. **(E)** Distribution of γH2A foci for each cell line. Cells are categorized in whole nucleus staining, ≥1 focus and without focus. **(F)** Plot shows the percentage of γH2A positive cells for each phase of the cell cycle: G1 or 1N1K; S or 1N1K* and 1N2K; and G2/M or 2N2K. **(G)** Distribution of γH2A foci pattern along the different phases (G1, early S, late S and G2/M) for each cell line. The asterisks show significant differences *vs* the parental line, calculated by the Student’s t test. *p < 0.05, **p < 0.01, ***p < 0.001. Bars, 5 μm.

Correct dNTP homeostasis is essential for adequate DNA replication and integrity (Kohnken et al., 2015). Nucleotide requirements differ during cell cycle progression and impaired dNTP levels at the onset of S phase can activate replication stress signaling (Forey et al., 2020). To investigate whether the loss or overexpression of TbHD82 can influence cell cycle progression, we performed a FACS analysis and measured N/K patterns in order to establish the percentage of cells in the G1, S phase, late S/G2 phase and mitosis as previously described (Marin et al., 2018). The data summarized in **Figure 4A** show that FACS analysis evidenced a small yet significant reduction in S phase and an increase in G1 cells in *Tb* BF *HD82*-dKO mutants. Furthermore, DAPI staining (**Figure 4B**) clearly illustrates an accumulation of 1N1K (G1 phase cells) and a decrease in 1N1K* (S phase cells) and 2N2K (G2/M phase) cells suggesting defects in the progression through S phase as a consequence of the absence of HD82 and the subsequent imbalance in dNTP pools.

**Figure 5:**
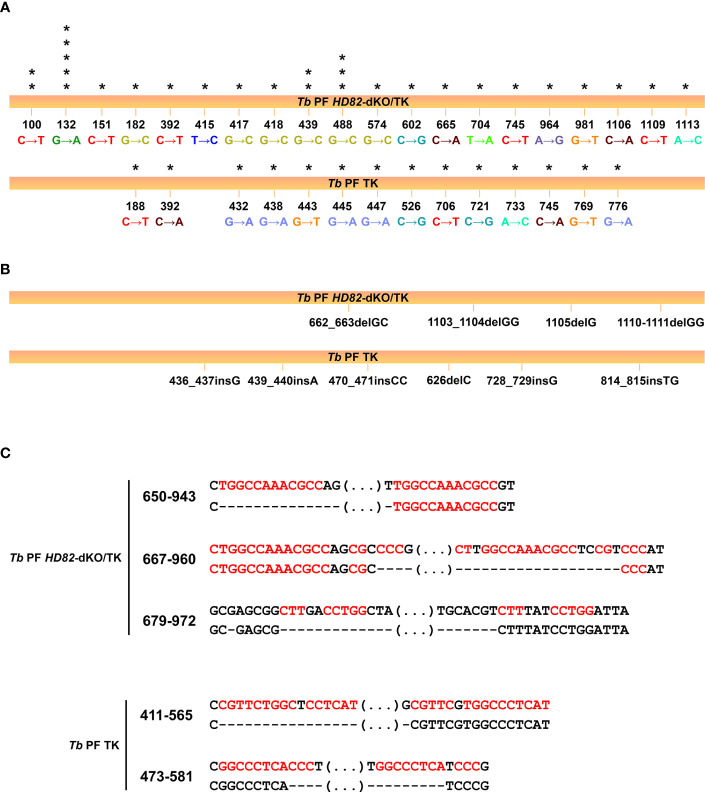
Mutation spectra obtained for *TK^−^* mutants isolated from the PF *HD82*-dKO/TK and PF/TK cell lines. **(A)** Occurrence and type of identified mutations in each cell line and their basepair position in the *TK* gene. Asterisks indicate the number of clones showing the mutation. **(B)** Short deletions and insertions observed in different revertant clones. **(C)** Base sequences flanking the large deletions observed in both cell lines. Each deletion corresponds to a different clone. Upper lines correspond to the theoretical *TK* sequence while lower lines denote the sequences found in the revertant clones. The dashed lines represent the beginning and the end of the deletions and the ellipses refer to regions that are not specifically shown. The repeat sequences are shown in red and numbers indicate the position of the deleted sequences.

It is well documented that establishing dNTP levels within a range that is optimal for adequate chromosomal replication is a crucial factor in maintaining genome stability (Pai and Kearsey, 2017). Since TbHD82 is important for dNTP homeostasis, we aimed to elucidate its potential role in genomic integrity. First, we performed a mutation rate assay based on the Luria-Delbrück test as described in Valdes et al. (1996) (Valdes et al., 1996; Castillo-Acosta et al., 2012b). For this assay we used *Tb* PF and *Tb* PF *HD82*-dKO cell lines expressing the thymidine kinase (*TK*) gene from Herpes simplex type 1 virus (HSV-1). As shown in **Table 1**, a 3.8-fold increase in the mutation rate was obtained for *Tb* PF *HD82*-dKO compared to *Tb* PF. The mutation spectra (**Table 2**, **Figure 5**) evidenced a notable increase in all types of mutations although GC to CG transversions (G→C and C→G), GC to AT transitions (G→A and C→T) and deletions were the most significantly increased (8.9, 4.4 and 7.9-fold, respectively).

**Table 1:** Experimental data for the Luria–Delbrück fluctuation analysis of reversion in TK transformed *T. brucei* PF lines.

TK transformant	PF TK	PF *HD82-*dKO/TK
Initial number of trypanosomes, Ni	200	200
Final number of trypanosomes, Nf	1.1 ± 0.1 x 10^6^	0.93 ± 0.02 x 10^6^
Total number of cell generations per culture, (Nf - Ni)/ln 2	1.5 ± 0.2 x 10^6^	1.34 ± 0.02 x 10^6^
Number of experimental cultures	96	96
Cultures without growth	66 ± 2	27 ± 3
Proportion of wells without cell growth, *P*_0_	0.68 ± 0.02	0.28 ± 0.03
Average number of mutants per culture, m = -ln *P*_0_	0.38 ± 0.02	1.3 ± 0.1
Mutation rate, m/total number of generations (cell/generation)	2.5 ± 0.2 x 10^-7^	9.4 ± 0.7 x 10^-7***^

The Luria–Delbrück fluctuation test was used to calculate the mutation rate to ganciclovir resistance for HSV-1 TK transfectants. The equation used is described in Materials and Methods.

*Results are represented as mean ± SD of three independent biological replicates. The asterisks show significant differences vs the parental line, calculated by the Student’s t test. ***p < 0.001.

**Table 2:** Spectra of Herpes simplex virus *TK* gene mutations.

Genotype	Mutation	Occurrence	Mutation Rate (10^-7^)^a^
**PF TK**	GC to TA	4/23 (17%)	0.41
	GC to CG	3/23 (13%)	0.31
	GC to AT	7/23 (30%)	0.69
	AT to CG	1/23 (4%)	0.09
	TA to AT	–	–
	AT to GC	–	–
	(-1) deletion	1/23 (4%)	0.09
	deletions	2/23 (9%)	0.21
	(+1) insertion	3/23 (13%)	0.31
	insertions	2/23 (9%)	0.20
**PF *HD82*-dKO/TK**	GC to TA	2/34 (5.9%)	0.55 [1.3] ^b^
	GC to CG	10/34 (29.4%)	2.76 [8.9] ^b^
	GC to AT	11/34 (32.3%)	3.04 [4.4] ^b^
	AT to CG	1/34 (2.9%)	0.28 [3.1] ^b^
	TA to AT	1/34 (2.9%)	0.28
	AT to GC	2/34 (5.9%)	0.55
	(-1) deletion	1/34 (2.9%)	0.28 [3.1] ^b^
	deletions	6/34 (17.6%)	1.66 [7.9] ^b^
	(+1) insertion	–	–
	insertions	–	–

a Mutation rates are the product of the proportion of a mutation specific class and the total mutation rate for each strain. In this experiment, the overall mutation rate for PF of parental and HD82-dKO strains were of 2.31 x 10^-7^ (Castillo-Acosta et al., 2012b) and 9.4 x 10^-7^ per cell generation, respectively. ^b^Number in brackets is the fold induction of a specific class of mutation relative to the parental line.

Subsequently, we investigated the presence of DNA repair foci in both parental and knockout BF cells by immunofluorescence (**Figure 4C**). The *Tb* BF *HD82*-cdKO cell line was also included in the analysis. For this purpose, we quantified the phosphorylation of Thr130 of trypanosomal histone H2A (known as γH2A), an early event that takes place in the DNA damage response involved in double strand break (DSB) repair (Glover and Horn, 2012). The percentage of γH2A positive cells was determined by fluorescence microscopy, evidencing a significantly increased staining for *Tb* BF *HD82*-dKO cells compared to the parental cell line (37% *vs* 24%, respectively), while induction of enzyme expression in the *Tb* BF *HD82*-cdKO mutant for 2 days reduced the number of positive cells from 42% in uninduced to 32% in induced cells (**Figure 4D**). The differential expression pattern of γH2A among cells (one focus, two or more foci or whole nucleus) was also determined and revealed that cells with whole nuclear staining were more abundant when TbHD82 was absent (**Figure 4E**). Likewise, when the cell cycle is considered (by DAPI staining), positive cells were mainly in the G1 and S phases which agrees with the concept that major DSB occurs during DNA replication and that repair probably occurs predominantly during the late S or G2 phases through the homologous recombination (HR) pathway (Marin et al., 2018). In agreement with the postulated defects in progression through the S phase, the null mutant exhibits a significant increase in G1 phase positive cells; 27% and 22% for *Tb* BF *HD82*-dKO and *Tb* BF *HD82*-cdKO -DOX *vs* 10% and 9% for *Tb* BF and *Tb* BF *HD82*-cdKO +DOX cells, respectively (**Figure 4F**). Furthermore, **Figure 4G** shows the percentage of cells in each phase with discrete foci or whole nucleus pattern, clearly indicating that whole nucleus marked cells are predominantly found in late S phase. On the other hand, positive cells in early S cells mainly show discrete foci. In summary, defects in cell cycle progression and increased damage are found upon elimination of TbHD82 in BF cells.

### TbHD82 expression is up-regulated upon genotoxic insult

**Figure 6:**
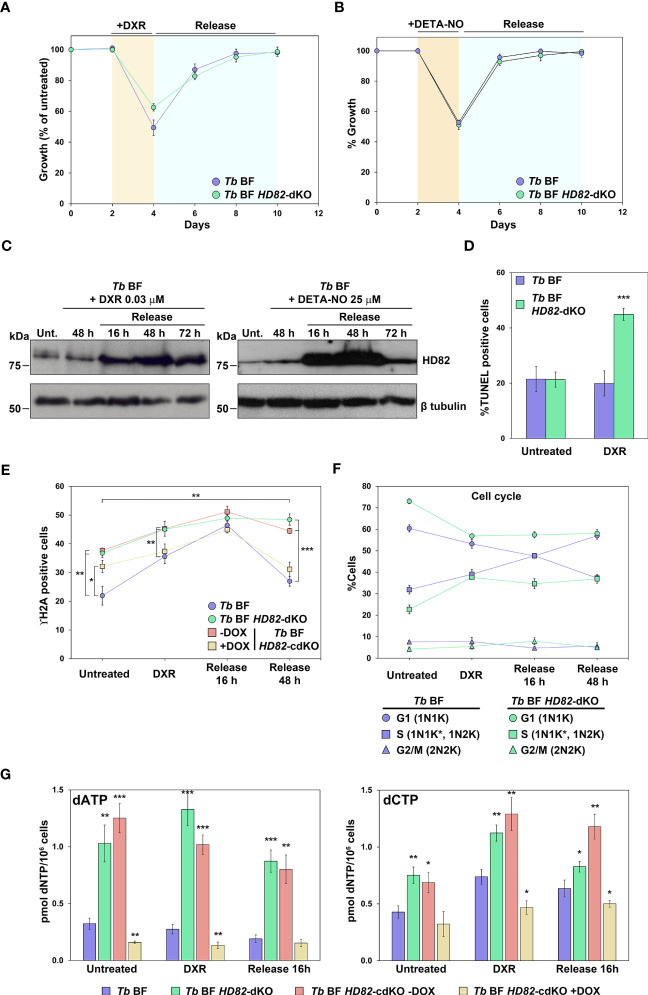
TbHD82 is involved in the DNA damage response induced by the genotoxic agents doxorubicin (DXR) and DETA-NO. **(A)** Graph displaying the growth of parental and null mutants when exposed to DXR for 48 h, followed by withdrawal after washing. **(B)** Proliferation of *Tb* BF and *Tb* BF *HD82*-dKO cells was studied during 48 h of 25 µM DETA-NO treatment and following withdrawal of the drug. **(C)** Western blotting of the time course for DXR and DETA-NO treatment in the parental cell line showing the upregulation of TbHD82 when parasites are exposed to the drugs. The HD82 expression profile was probed using the anti-TbHD82 antibody and normalized using anti-Tbβ-tubulin as loading control. **(D)** The percentages of TUNEL positive parental and HD82-deficient cells after DXR treatment are illustrated. **(E)** Total γH2A positive cells were monitored during treatment and removal in *Tb* BF, *Tb* BF *HD82*-dKO as well as in *Tb* BF *HD82*-cdKO ± DOX parasites. **(F)** Time course of cell cycle progression evaluated by DAPI staining during DXR treatment as well as after withdrawal. *Tb* BF and *Tb* BF *HD82*-dKO cells were classified according to the number of nuclei (N) and kinetoplasts (K): 1N1K (G1 phase); 1N1K* (asterisk denotes an enlarged K under segregation) and 1N2K (S-phase); and 2N2K (G2/M phase). **(G)** dATP and dCTP levels were monitored upon DXR exposition and after 16 h of agent removal in *Tb* BF, *Tb* BF *HD82*-dKO as well as *Tb* BF *HD82*-cdKO in both absence or presence of DOX parasites. Data are presented as the mean percentage (± SD) of total cells counted from three independent experiments (n > 150 cells in total). The asterisks in panels D and G show significant differences *vs* the parental line, calculated by the Student’s t test. The asterisks at vertical lines in panel E show the significant differences between *Tb* BF *HD82*-dKO and *Tb* BF *HD82*-cdKO ± DOX *vs* the parental line, while the asterisks at horizontal lines indicate the significant differences, for both the *Tb* BF *HD82*-dKO and *Tb* BF *HD82*-cdKO – DOX cell lines, between release for 48 h and untreated conditions, calculated by the Student’s t test. *p < 0.05, ** *p* < 0.01, ***p < 0.001.

Previous studies have suggested that dNTP triphosphohydrolases such as human SAMHD1 could be involved in repair mechanisms and recruiting repair factors to DSB sites (Daddacha et al., 2017; Coquel et al., 2018). Moreover, in human cells, SAMHD1 has been shown to have an important role in the response and recovery from stress when exposed to ROS (Kapoor-Vazirani et al., 2022). Hence, we aimed to evaluate the behavior of TbHD82 upon genotoxic insult. Once inside the mammalian host, trypanosomes are in contact with oxidative and genotoxic stress as a consequence of the immune response (Mabbott and Sternberg, 1995; Figarella et al., 2018). We first evaluated the proliferation of *TbHD82* null mutants upon exposure to the genotoxic agents doxorubicin (DXR), diethylenetriamine-nitric oxide (DETA-NO, an exogenous nitric oxide donor), and the oxidizing agent hydrogen peroxide (H_2_O_2_). Subtle yet significant increases in EC_50_ (**Table 3**) were denoted in knockout compared to parental cells after treatment with DXR (**Figure S5A**) and H_2_O_2_ (**Figure S5C**) while no significant changes in EC_50_ values were observed with DETA-NO (**Figure S5B**). DXR is a DNA intercalating agent that causes breakage of DNA strands and inhibition of both DNA and RNA synthesis. DXR inhibits the enzyme topoisomerase II, causing DNA damage and induction of apoptosis (Wassermann et al., 1990). Despite the minor changes in EC50 values, further TUNEL analysis revealed that *Tb* BF *HD82*-dKO cells treated with 0.03 µM DXR exhibit significantly increased damage (2.3-fold) compared to parental cells (**Figure 6D**). We subsequently performed treatment and recovery assays in *Tb* BFs; cells were treated for 48 h with either 0.03 µM DXR or 25 µM DETA-NO and then cultures were washed to remove the genotoxic agent and allowed to recover for 72 h. Interestingly increased TbHD82 expression was observed during recovery for both DXR (5.2-fold) (**Figures 6A, C**) and DETA-NO (9.0-fold) (**Figures 6B, C**).

**Table 3:** EC_50_ values for DXR, DETA-NO and H_2_O_2_ in HD82-deficient and parental parasites.

Cell lines	EC_50_ DXR (µM)	EC_50_ DETA-NO (µM)	EC_50_ H_2_O_2_ (µM)
*Tb* BF	0.032 ± 0.002	23.2 ± 0.2	410 ± 30
*Tb* BF *HD82*-dKO	0.042 ± 0.002 (***)	24.1 ± 0.2	640 ± 120 (*)

Results are represented as mean ± SD of three independent biological replicates. The asterisks show significant differences vs the parental line, calculated by the Student’s t test. *p < 0.05 and ***p < 0.001.

This observation was analyzed in detail in the case of DXR exposure. First, when DNA repair was quantified by determining γH2A, we observed that parental cells 48 h after wash-out, present a similar number of γH2A positive cells than prior to treatment indicating effective recovery (**Figure 6E**). On the other hand, in null mutants recovery is less efficient suggesting that the absence of HD82 is detrimental to effective repair after genotoxic insult. It must be noted that during recovery of parental cells (16 h) there is a significant increase in the number of cells in S phase (observed by DAPI staining) undergoing active replication which might partially explain the increased levels of TbHD82 whose expression, as previously indicated, is maximum in S phase (**Figures 6F**, **S6A**). When dNTP levels were measured in *Tb* BF cells treated with DXR and upon wash-out, surprisingly, increased TbHD82 does not result in diminished dNTPs; rather dCTP is somewhat increased upon DXR treatment. In null mutants, dNTP levels remain increased during treatment and recovery (**Figures 6G**, **S6B**).

## Discussion

The equilibrium of the dNTP pool is finely regulated during the cell cycle and DNA replication. However, dNTP pool homeostasis is not only essential for replication but also has an important role in DNA damage repair and recombination. Thus, a balanced level of dNTPs in eukaryotic cells are crucial for the fidelity of DNA synthesis and genome stability (Franzolin et al., 2013). In trypanosomes the synthesis of dNTPs has been documented to occur mainly via*de novo* pyrimidine synthesis and pyrimidine and purine salvage and subsequent action of ribonucleotide reductase (Hofer et al., 1997). The degradation of dNTPs has been less studied and dNTPases responsible for direct hydrolysis of dNTPs into dNs and triphosphates have been poorly characterized with the exception of TbHD52, a mitochondrial orthologue of TbHD82 involved in dCTP and dTTP homeostasis (Yague-Capilla et al., 2021). In human cells, the major dNTPase, SAMHD1, contains an N-terminal sterile α motif (SAM) domain and a central histidine-aspartic (HD) domain. The SAM domain is a protein-protein and protein-nucleic acid interaction module while the HD domain has dNTPase or phosphohydrolase activity (Goldstone et al., 2011; White et al., 2013). Human SAMHD1 presents a major nuclear localization (Brandariz-Nuñez et al., 2012) albeit undergoes nucleocytoplasmic shuttling (Du et al., 2019).

TbHD82 contains an HD domain and was predicted to be a triphosphohydrolase, member of the SAMHD1 family of proteins. While a SAM domain is absent, it is possible that the N-terminal and C-terminal extensions observed in the trypanosomal protein may account for a similar function. Since using both specific antibodies and overexpression of a c-myc-tagged protein, TbHD82 was found to be present in the nucleus and on the other hand, protein sequence alignment showed that 15 of the 16 amino acids constituting the substrate binding site and residues involved in catalysis were conserved (**Figure S7**), it is highly probable that the protein also possesses dNTPase activity.

Substrate synthesis and degradation, DNA synthesis and repair, and nucleoside import and export form a controlled regulatory scheme that maintains homeostatic dNTP concentrations inside the cell. The levels must be exquisitely tuned depending on the cell type and stage during the cell cycle. In both bloodstream and procyclic trypanosomes, the lack of TbHD82 results in elevated levels of dCTP, dATP and dTTP denoting a role for the enzyme in dNTP metabolism. Each canonical dNTP is not equally abundant within the cell (Huang et al., 2020). In trypanosomes we found dCTP to be the most abundant in parental parasites and dATP the most markedly increased upon TbHD82 elimination. While dGTP appeared to be the only dNTP non-sensitive to TbHD82 depletion, overexpression of the enzyme in the *Tb* BF *HD82*-cdKO resulted in slightly diminished levels. It has been described that SAMHD1 shows different affinities for each of the four canonical dNTPs (Cardamone et al., 2017). While it is possible that the catalytic efficiency of TbHD82 for dGTP is lower, additional mechanisms might be involved in the modulation of dGTP levels in null mutants such as decreased GDP reduction or increased degradation.

In summary, the measurement of dNTP levels suggests that TbHD82 has a critical role in the maintenance of dNTP pools. Despite multiple efforts, active recombinant active protein was not achievable and the identification of the specific substrates and kinetic parameters has not been possible. In any case the data here presented firmly points towards a central role of the enzyme in dNTP hydrolysis.

While dNTP concentrations are asymmetric, the proper balance of the dNTP pool is crucial to cell survival and genomic integrity (Meuth, 1989). Imbalanced dNTP pools, resulting from DNA damage, perturbations of metabolism, or mutations in enzymes involved in maintaining the dNTP equilibrium, can generate detrimental consequences that include increased mutation rates and the induction of a mutator phenotype, DNA damage and activation of the DNA damage response, replication stress, and stalled replication forks and deficient cell cycle progression (Ke et al., 2005; Chabes and Stillman, 2007; Hastak et al., 2008; Jasencakova et al., 2010; Kumar et al., 2010; Kumar et al., 2011; Davidson et al., 2012; Buckland et al., 2014; Gemble et al., 2016; Watt et al., 2016). In most cell types SAMHD1 is constitutively expressed at high levels in both cycling and non-cycling myeloid and lymphoid cells, including monocytes, macrophages, dendritic cells, and CD4+ T-cells, although its activity can be differentially regulated by post-translational modification (Cribier et al., 2013; Schmidt et al., 2015; Yan et al., 2015). Here we found that expression of TbHD82 was maximum in S phase cells and null mutants present defects in S phase progression with an accumulation of cells in G1. In a similar fashion, although constitutively expressed, SAMHD1 is widely established as a central regulator of dNTP pool dynamics within cells (Kim et al., 2012; Franzolin et al., 2013) and silencing or degradation of SAMHD1 results in increased dNTP pools and altered growth kinetics as evidenced by studies that demonstrate an increased retention of cells in the G1 phase with a corresponding loss in S phase cells (Franzolin et al., 2013; Kretschmer et al., 2015; Bonifati et al., 2016). In yeast *Saccharomyces cerevisiae*, constitutively high dNTP concentrations inhibit cell cycle progression and the DNA damage checkpoint (Chabes and Stillman, 2007). We propose that also in *Trypanosoma*, fluctuation of dNTP concentration controls cell cycle progression and the initiation and progression of DNA replication.

As previously mentioned, adequate dNTP concentrations are also essential for genomic stability. High dNTP levels are detrimental to the fidelity of DNA replication in bacteria (Gon et al., 2011), yeast (Chabes et al., 2003; Fleck et al., 2013) and mammalian cells (Weinberg et al., 1981; Caras and Martin, 1988). This suggests, at least partially, the tendency of DNA polymerases to extend a mismatched primer-template and the lower efficiency of proofreading when nucleotide levels are enhanced (Beckman and Loeb, 1993; Kunkel and Bebenek, 2000). In *S. cerevisiae*, very high levels of dNTPs resulting from overexpression of a ribonucleotide reductase (RNR) variant not subject to dATP inhibition (D57N mutation in the large subunit) causes a delay to S phase entry that appears to act before the Cdc45 loading step at initiation (Chabes and Stillman, 2007). Increasing dNTP levels also reduces the length of S phase under unstressed conditions, suggesting that physiological nucleotide levels are limiting for DNA synthesis, a discovery which is in agreement with the analysis of DNA synthesis rates *in vitro* (Kunkel et al., 1987; Stodola and Burgers, 2016).

In line with this concept, in *Tb HD82*-dKO mutants the expanded dNTP pools appear to not only hinder S phase progression but also activate the DNA damage response. In general, endogenous DSBs can be due to metabolic reactions or genotoxic agents. For instance, endogenous DSBs can arise during the attempted repair of oxidized DNA bases (Yang et al., 2004; Cannan et al., 2014); or during DNA replication due to situations that may lead to pausing or blocking of the replication fork (Mirkin and Mirkin, 2007; García-Muse and Aguilera, 2016; da Silva et al., 2019); or during the repair of single-stranded DNA breaks (SSBs) generated in the S phase (Vilenchik and Knudson, 2003; Saleh-Gohari et al., 2005; Elango et al., 2017). Previous studies have suggested that endogenous levels of DNA lesions represented by γH2A fluorescent foci in *T. brucei*, an early response to the induction of DNA damage, accumulate predominantly throughout the G1, S, and G2 phases probably due to the fact that, when DNA lesions occur (during the S phase), their repair takes place predominantly in the late S or G2 phases via the HR pathway (Marin et al., 2018). When measuring H2A phosphorylation we evidenced that for both parental and mutant cells, indeed foci accumulate mostly during the G1 and S phases (**Figure 4F**). In addition, the relative number of cells with repair foci was significantly increased in null mutants. When analyzed in detail, the relative amount of G1 foci positive cells was enhanced in KO cells, in agreement with the indicated defects in S phase progression and subsequent accumulation of cells in G1.

The mutation rate and spectra analyses were performed in procyclic forms and compared to previous data (Castillo-Acosta et al., 2012b). A 3.8-fold increase in the mutation rate to ganciclovir resistance in null mutants and the analysis of the type of mutations would support the observation that the dNTP imbalance in KO cells affects the overall mutation spectra. For instance, the significant increase in G:C to C:G transversions would be expected when dCTP is inserted opposite template C during replication since the dCTP/dGTP ratio is significantly enhanced in mutant cells (2-fold higher in HD82-deficient *vs* parental parasites). Likewise the transition G:C to A:T corresponding to G→A and C→T mutations (**Figure 5A**) may occur when dATP rather than dGTP is inserted opposite template C (the dATP/dGTP ratio is 10-fold higher in null mutants) or when dTTP rather than dCTP is inserted opposite template G (the dTTP/dCTP ratio is 1.6-fold higher in null mutants). It has been reported that sequence context also contributes to explain the nature of the mutations and the resulting mismatches remain at the expense of polymerase proofreading and mismatch repair (Buckland et al., 2014). It is known that DNA breaks stimulate HR. Additionally, large deletions in *E. coli dut* mutants were found to be flanked by repeated sequences (Sedwick et al., 1986). We have previously shown, dUTPase-deficient *T. brucei* parasites were prone to show large deletions, potentially as a consequence of DSB repair by HR and microhomology-mediated end joining, an event that uses 5-20 bp flanking microhomologies (Glover et al., 2008; Castillo-Acosta et al., 2012a). In the present study, large deletions show similar flanking regions, an observation that is consistent with microhomology-mediated end joining events.

In yeast DNA damage leads to a 6- to 8-fold increase in dNTP levels. This increase is conferred by an unusual, relaxed dATP feedback inhibition of RNR. The increase in dNTP pools dramatically improves survival following DNA damage, but at the same time leads to higher mutation rates (Chabes et al., 2003). In *Trypanosoma* only dCTP levels appear to increase upon DXR treatment in parental cells. The absence of TbHD82 provoked enhanced levels of dCTP, dATP and dTTP yet only dCTP was further increased upon exposure to the genotoxic agent. Surprisingly levels of TbHD82 in parental cells during treatment and release were enhanced 5.2-fold yet this up-regulation did not correlate with a decrease in dNTPs. It is probable that also in *Trypanosoma*, other mechanisms contribute to dNTP maintenance upon genotoxic insult such as an up-regulation of RNR activity and increased salvage (Chabes et al., 2003). In addition, it may be speculated that TbHD82 has extra roles in the DSB repair pathway. In this sense it has been reported that SAMHD1 has a dNTPase-independent function in promoting DNA end resection to facilitate DSB repair by HR. SAMHD1 deficiency causes hypersensitivity to DSB-inducing agents, and is recruited to DSBs. SAMHD1 complexes with CtIP via a conserved C-terminal domain and recruits CtIP to DSBs to facilitate end resection and HR (Daddacha et al., 2017). Whether TbHD82 has a similar function in trypanosomes remains to be established.

In summary, *TbHD82* null mutants, while displaying an unaltered growth profile, are defective in S phase progression, and exhibit a mutator phenotype and enhanced activation of the DNA damage response, features that are in line with the consequences of dNTP imbalances. We propose that the enzyme, by degrading dNTPs in both BF and PF cells, inhibits accumulation to levels that would impede the efficiency or correctness of the replication and repair machinery. Hence TbHD82 would act as a guardian of genomic fidelity by modulating excessive accumulation of undesirable dNTPs.

## Data availability statement

The raw data supporting the conclusions of this article will be made available by the authors, without undue reservation.

## Author contributions

Conceived and designed the experiments: DG-P, LR-P, VC-A, PA-P and CB-N. Performed the experiments: PA-P, VC-A and CB-N. Analyzed the data: DG-P, LR-P, VC-A, PA-P and CB-N. Contributed reagents/materials/analysis tools: DG-P, LR-P. Wrote the paper: DG-P, VC-A and PA-P. All authors contributed to the article and approved the submitted version.

## Supplementary material

Supplementary Figure 1 Three-dimensional modeling of the TbHD82 structure constructed by AlphaFold2 algorithm from Neurosnap web tool. Structure shows two disordered regions; the purple one corresponds to the amino acids between 641-675 and the yellow corresponds to the amino acids between 713-735. The central region is colored in red and correspond to the amino acids between 216-366. The predicted structure shows a Mean pLDDT and Max PAE scores of 72.13 and 31.5, respectively.

Supplementary Figure 2 Growth and HD82 expression analysis in both BF and PF cell lines overexpressing a native TbHD82 **(A, B)** and a c-myc tagged fusion protein (TbHD82-myc) **(C**, **D).** Proliferation was measured in three independent replicates. Western blotting was performed to check both native TbHD82 and c-myc tagged fusion protein (TbHD82-myc) expression using an anti-TbHD82 primary antibody. In addition, *Tb* HD82-myc cell lines were also probed with an anti-c-myc primary antibody. An HPRT-conjugated anti-rabbit secondary antibody illustrates the levels of TbHD82 in different parasites. TbHD82 signal was normalized using anti-Tbβ-tubulin as loading control. **(E)** Specificity of the affinity-purified rabbit polyclonal anti-TbHD82 antibody was evaluated by western blotting using BF and PF trypanosome extracts.

Supplementary Figure 3 Genotypic analysis of *HD82*-double KO (*HD82*-dKO) and conditional KO (*HD82*-cdKO) cells containing an inducible ectopic copy of *HD82* in both BF and PF. **(A)** *HD82* locus architecture (Gene DB database, Tb427.06.2900 as systematic name) and the corresponding two alleles of the blasticidin (*BSD*) and hygromycin (*HYG*) resistance genes after replacements. The ectopic copy of *HD82* under the control of the tetracycline-inducible promoter is also illustrated. Tb427.06.2890 (upstream) and Tb427.06.2910 (downstream) denote the open reading frames flanking the 5’UTR and 3’UTR of *HD82* gene. **(B**, **C)** The correct allelic replacement was verified by PCR using different oligonucleotides targeted against the *HD82* locus. *BSD* replacement was checked using VCA175/BSD 3-5 (a, 1227 bp) and VCA176/BSD 5-3 (b, 1288 bp) oligonucleotides, *HYG* replacement was checked using VCA175/VCA34 (c, 1756 bp) and VCA176/VCA33 (d, 1808 bp) oligonucleotides. dKO and PL show the additional PCR performed with specific oligonucleotides, VCA147 and VCA148, amplifying the open reading frame of the *HD82* gene (2221 bp) in HD82-deficient and parental (PL) cells, respectively.

Supplementary Figure 4 Proliferation of trypanosomes overexpressing TbHD82. Expression is under the control of a tetracycline-inducible promoter. Plots in left panels show the growth percentages in both BF **(A)** and PF **(B)** of uninduced and induced *Tb HD82*-cdKO parasites by DOX, compared to the growth of the corresponding parental line. Proliferation was measured in three independent biological replicates. Right panel, western blotting was performed to verify the expression of TbHD82 using the anti-TbHD82 primary antibody and HPRT-conjugated anti-rabbit secondary antibody. The TbHD82 signal was normalized using anti-Tbβ-tubulin as loading control.

Supplementary Figure 5 Effect of the genotoxic agents, DXR, DETA-NO and H_2_O_2_ on *Tb* BF *HD82*-dKO proliferation and HD82 expression. Dose response curves of *Tb* BF and *Tb* BF *HD82*-dKO parasites treated for 48 h with increasing concentrations of DXR **(A)** and DETA-NO **(B)**, or for 45 min with increasing concentrations of H_2_O_2_ **(C).**

Supplementary Figure 6 TbHD82 is involved in the DNA damage response induced by the genotoxic agent doxorubicin (DXR). **(A)** Uninduced and induced *Tb* BF *HD82*-cdKO parasites were used to analyze cell cycle progression evaluated by DAPI staining during DXR treatment and after withdrawal. Parasites were classified according to the number of nuclei (N) and kinetoplasts (K): 1N1K (G1 phase); 1N1K* (asterisk denotes an enlarged K under segregation) and 1N2K (S phase); and 2N2K (G2/M phase). **(B)** dGTP and dTTP levels were monitored upon DXR exposition and after 16 h of agent removal in *Tb* BF, *Tb* BF *HD82*-dKO as well as *Tb* BF *HD82*-cdKO ± DOX parasites. Data are presented as the mean percentage (± SD) of total cells counted from three independent experiments (n > 150 cells in total). The asterisks show significant differences vs the parental line, calculated by the Student’s t test. **p* < 0.05, ***p* < 0.01, ****p* < 0.001.

Supplementary Figure 7
**(A)** Comparison of protein length and the location of the HD domain between human SAMHD1 and TbHD82, with relevant amino acid positions marked. Prediction of the conserved domains was obtained with Pfam. **(B)** Alignment of TbHD82 with the representative HD containing proteins HsSAMHD1 and the dNTPase from *Enterococcus faecalis*. Alignment was carried out using ClustalOmega and displayed with JalView. Sequences were collected from UniProt database with the following protein IDs: HsSAMHD1 (Q9Y3Z3), EfdNTPase (Q836G9), TbHD82 (Q583P4) and TbHD52 (Q57X97). Asterisks indicate amino acids involved in catalysis and substrate binding: in red, residues involved in ribose exclusion; in black, residues involved in α-phosphate binding; in green, HD residues; in purple, catalytic residues; in blue, residues involved in Mg^2+^ coordination with the β and γ-phosphate groups.
