# Supplementary figures and images for: A nuclear orthologue of the dNTP triphosphohydrolase SAMHD1 controls dNTP homeostasis and genomic stability in Trypanosoma brucei
Source: Front Cell Infect Microbiol. 2023 Aug 22;13:1241305. doi: 10.3389/fcimb.2023.1241305 (PMC10478004; doi:10.3389/fcimb.2023.1241305)

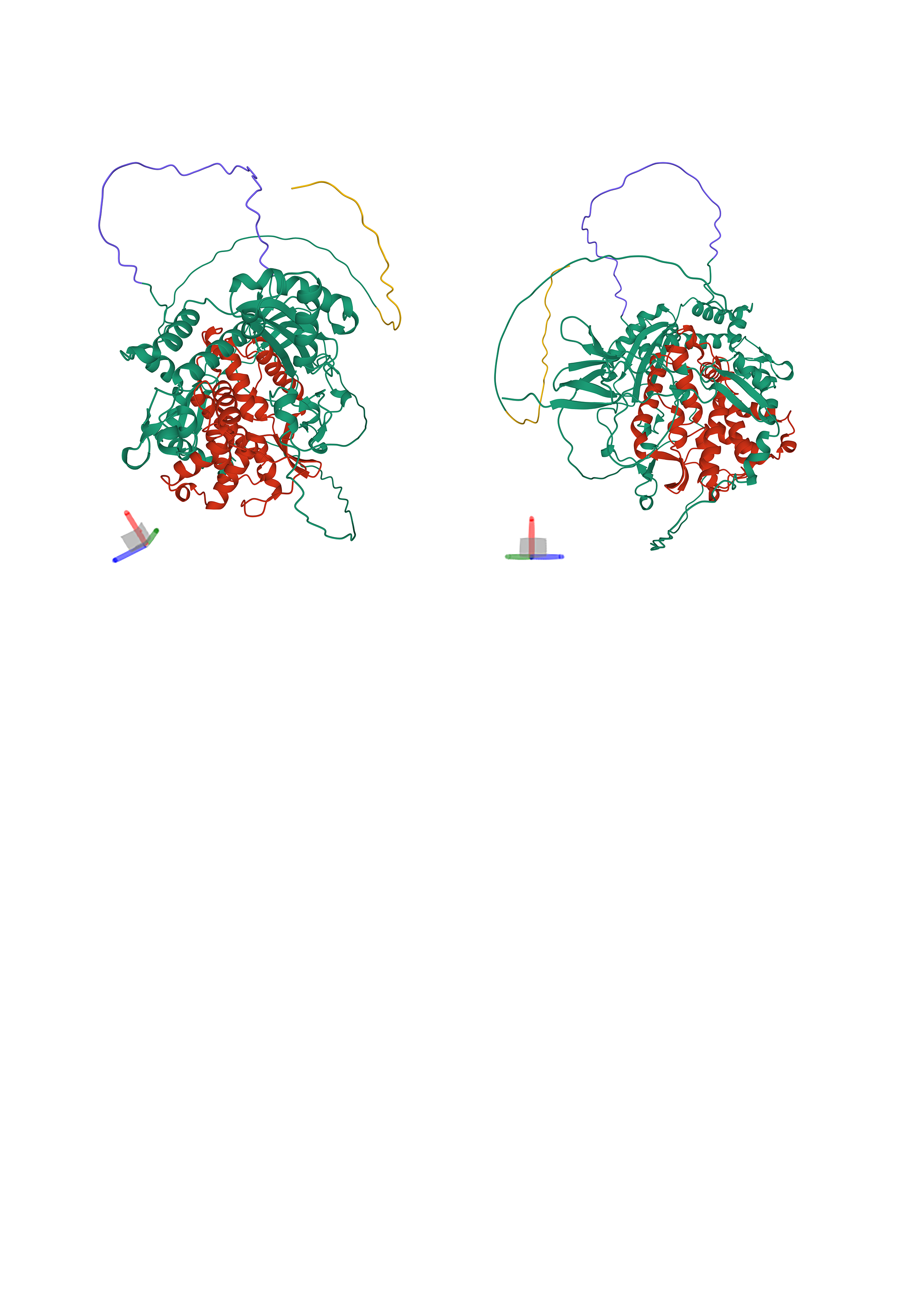

Supplement: Supplementary Figure 1 — Three-dimensional modeling of the TbHD82 structure constructed by AlphaFold2 algorithm from Neurosnap web tool. Structure shows two disordered regions; the purple one corresponds to the amino acids between 641-675 and the yellow corresponds to the amino acids between 713-735. The central region is colored in red and correspond to the amino acids between 216-366. The predicted structure shows a Mean pLDDT and Max PAE scores of 72.13 and 31.5, respectively. [file Image_1.tif]

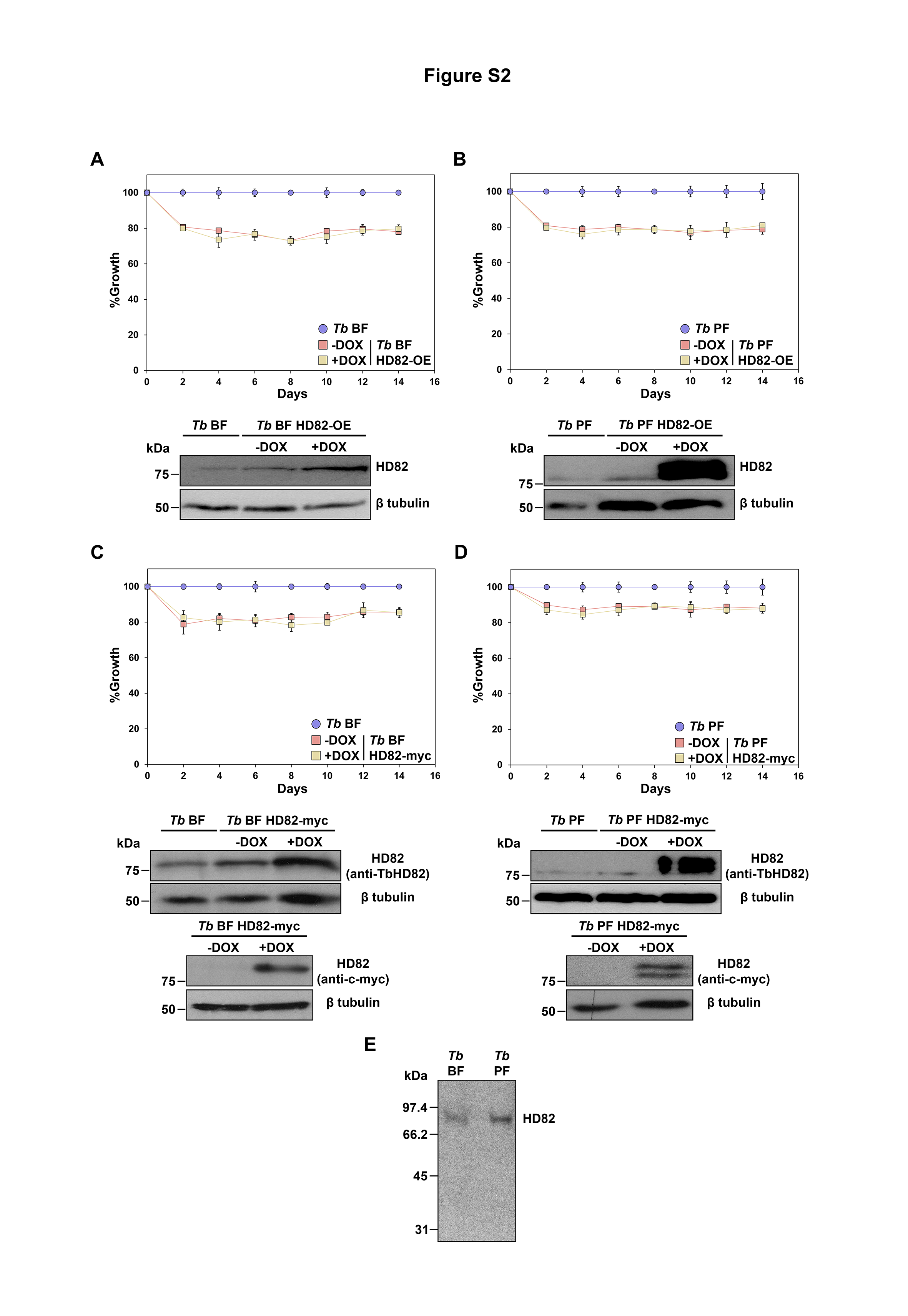

Supplement: Supplementary Figure 2 — Growth and HD82 expression analysis in both BF and PF cell lines overexpressing a native TbHD82 (A, B) and a c-myc tagged fusion protein (TbHD82-myc) (C, D). Proliferation was measured in three independent replicates. Western blotting was performed to check both native TbHD82 and c-myc tagged fusion protein (TbHD82-myc) expression using an anti-TbHD82 primary antibody. In addition, Tb HD82-myc cell lines were also probed with an anti-c-myc primary antibody. An HPRT-conjugated anti-rabbit secondary antibody illustrates the levels of TbHD82 in different parasites. TbHD82 signal was normalized using anti-Tbβ-tubulin as loading control. (E) Specificity of the affinity-purified rabbit polyclonal anti-TbHD82 antibody was evaluated by western blotting using BF and PF trypanosome extracts. [file Image_2.tif]

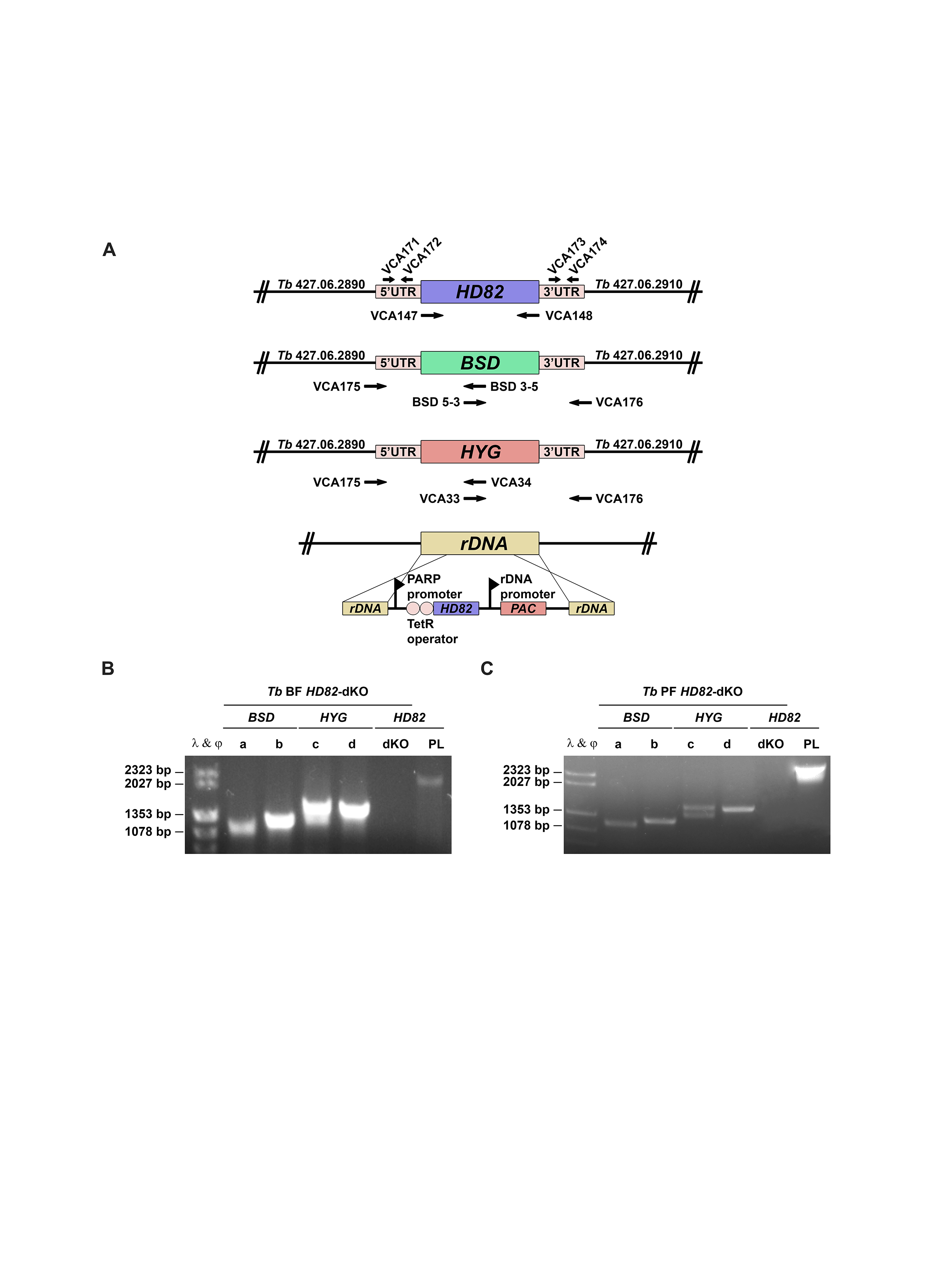

Supplement: Supplementary Figure 3 — Genotypic analysis of HD82-double KO (HD82-dKO) and conditional KO (HD82-cdKO) cells containing an inducible ectopic copy of HD82 in both BF and PF. (A) HD82 locus architecture (Gene DB database, Tb427.06.2900 as systematic name) and the corresponding two alleles of the blasticidin (BSD) and hygromycin (HYG) resistance genes after replacements. The ectopic copy of HD82 under the control of the tetracycline-inducible promoter is also illustrated. Tb427.06.2890 (upstream) and Tb427.06.2910 (downstream) denote the open reading frames flanking the 5’UTR and 3’UTR of HD82 gene. (B, C) The correct allelic replacement was verified by PCR using different oligonucleotides targeted against the HD82 locus. BSD replacement was checked using VCA175/BSD 3-5 (a, 1227 bp) and VCA176/BSD 5-3 (b, 1288 bp) oligonucleotides, HYG replacement was checked using VCA175/VCA34 (c, 1756 bp) and VCA176/VCA33 (d, 1808 bp) oligonucleotides. dKO and PL show the additional PCR performed with specific oligonucleotides, VCA147 and VCA148, amplifying the open reading frame of the HD82 gene (2221 bp) in HD82-deficient and parental (PL) cells, respectively. [file Image_3.tif]

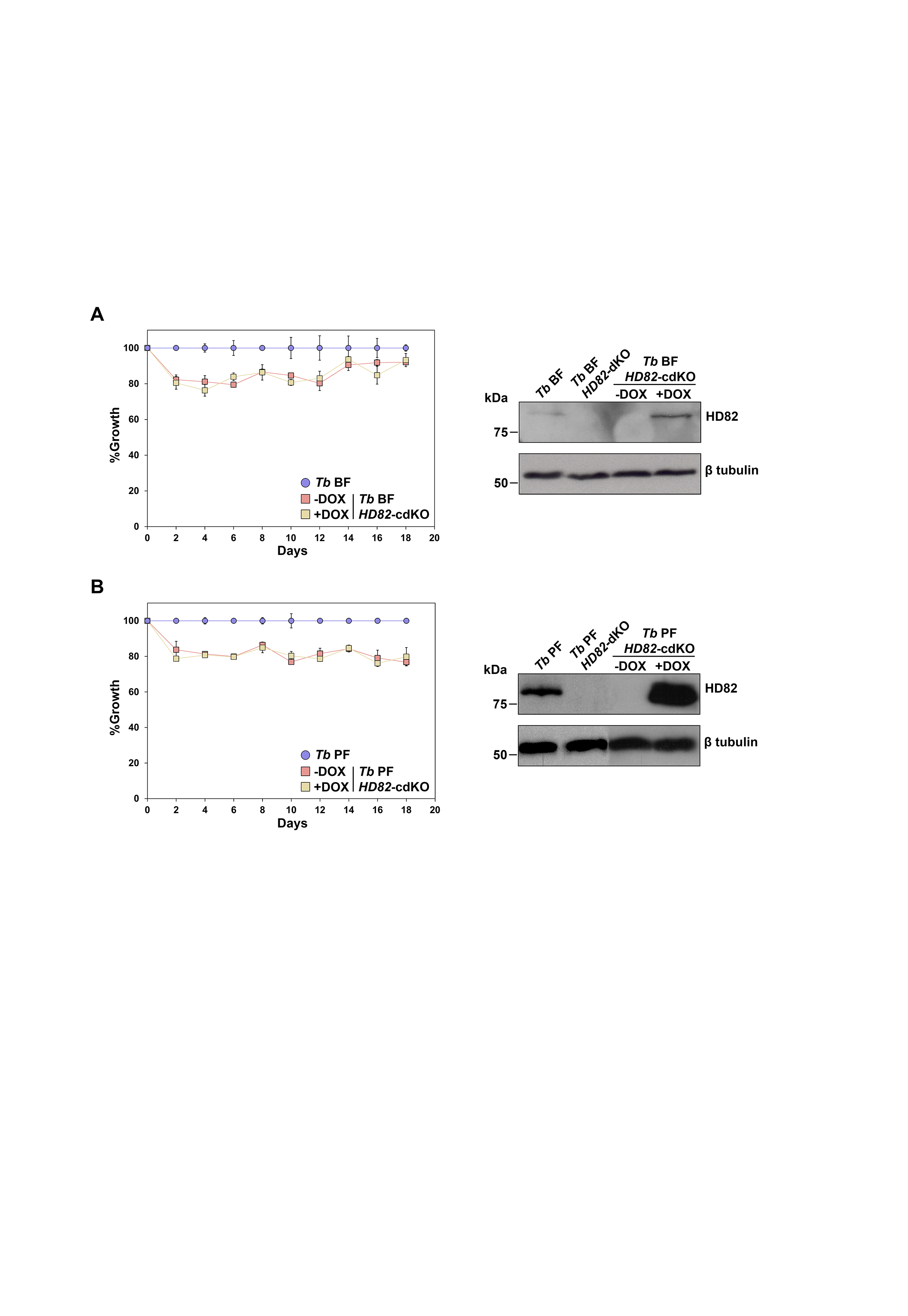

Supplement: Supplementary Figure 4 — Proliferation of trypanosomes overexpressing TbHD82. Expression is under the control of a tetracycline-inducible promoter. Plots in left panels show the growth percentages in both BF (A) and PF (B) of uninduced and induced Tb HD82-cdKO parasites by DOX, compared to the growth of the corresponding parental line. Proliferation was measured in three independent biological replicates. Right panel, western blotting was performed to verify the expression of TbHD82 using the anti-TbHD82 primary antibody and HPRT-conjugated anti-rabbit secondary antibody. The TbHD82 signal was normalized using anti-Tbβ-tubulin as loading control. [file Image_4.tif]

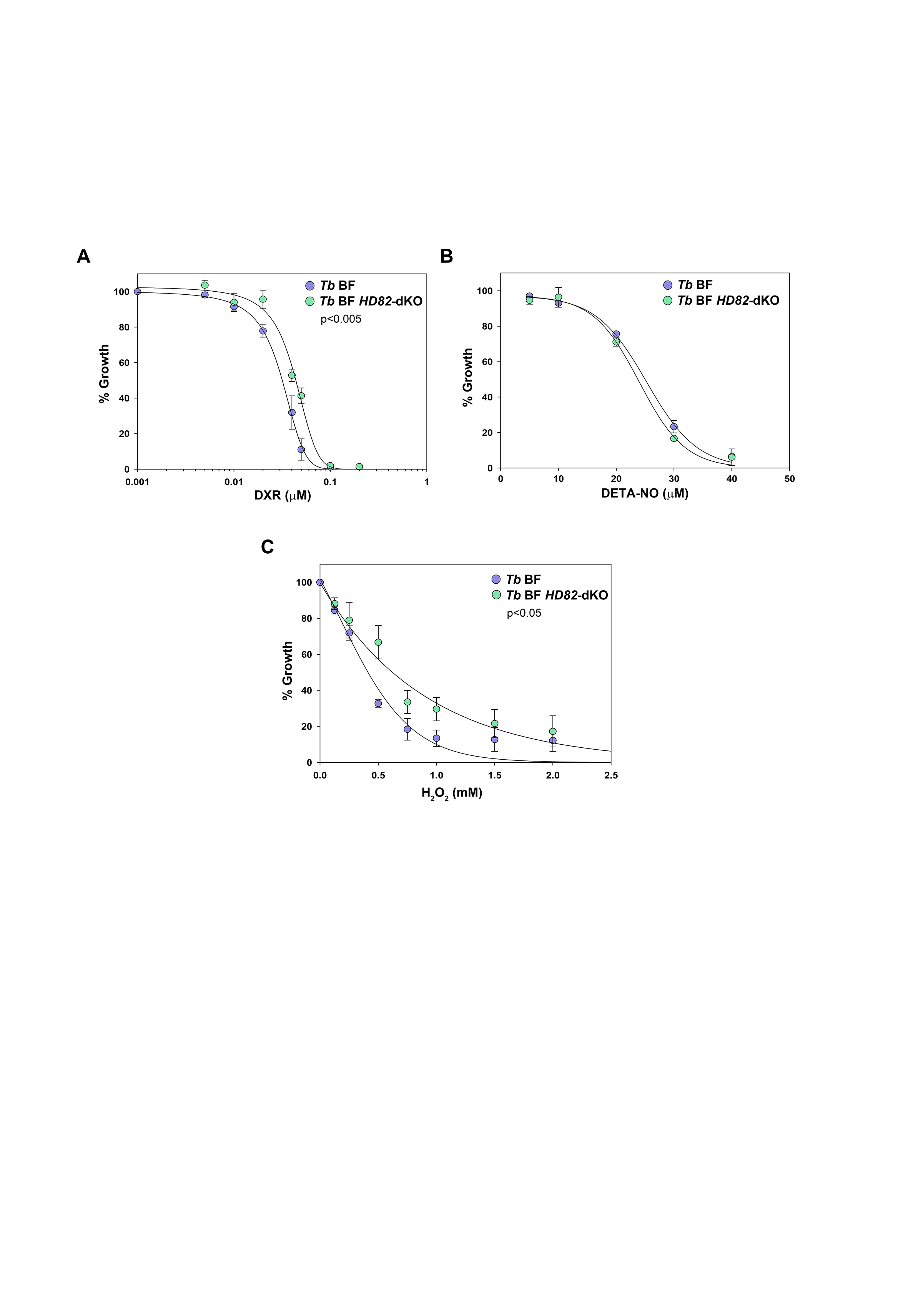

Supplement: Supplementary Figure 5 — Effect of the genotoxic agents, DXR, DETA-NO and H2O2 on Tb BF HD82-dKO proliferation and HD82 expression. Dose response curves of Tb BF and Tb BF HD82-dKO parasites treated for 48 h with increasing concentrations of DXR (A) and DETA-NO (B), or for 45 min with increasing concentrations of H2O2 (C). [file Image_5.tif]

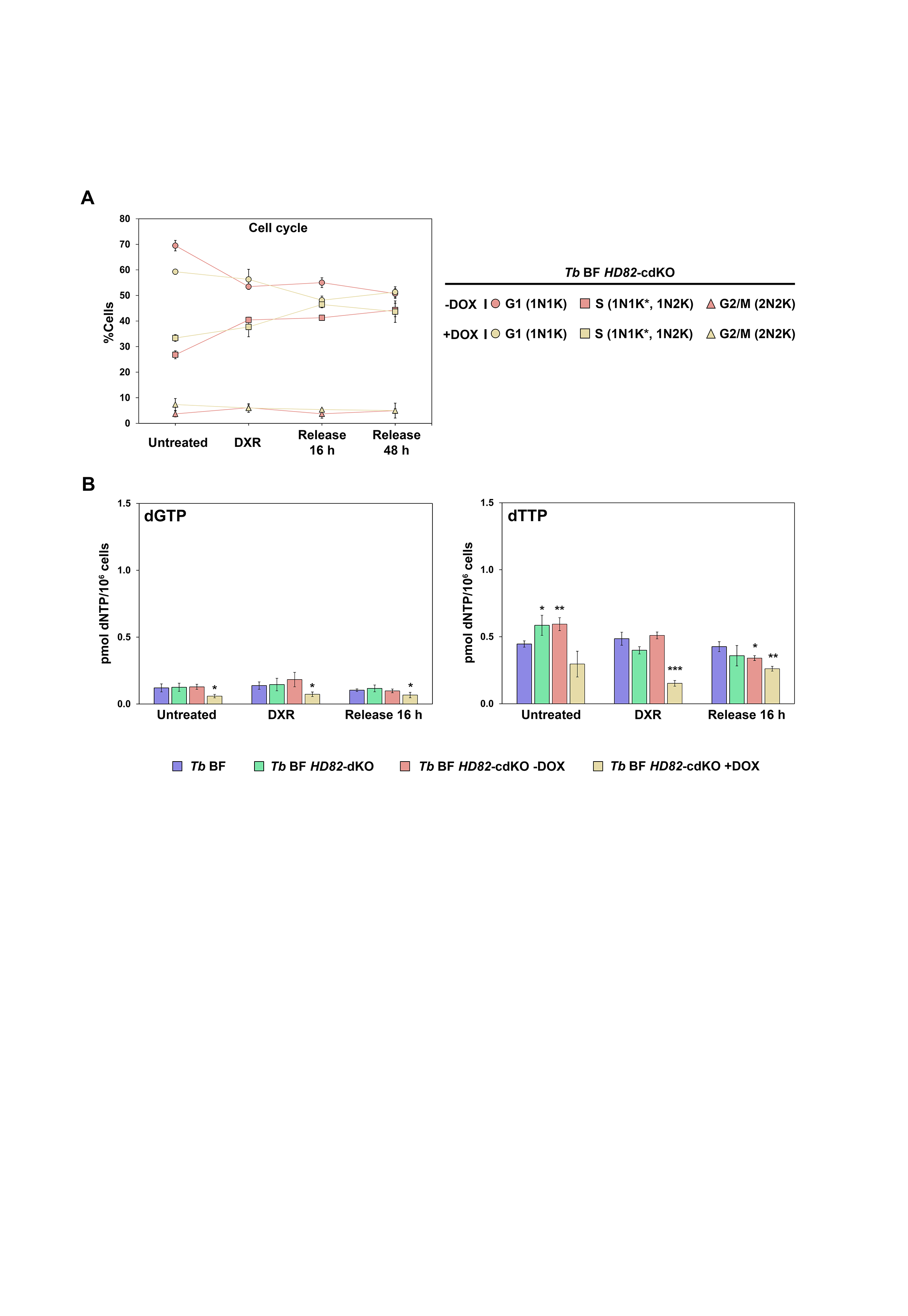

Supplement: Supplementary Figure 6 — TbHD82 is involved in the DNA damage response induced by the genotoxic agent doxorubicin (DXR). (A) Uninduced and induced Tb BF HD82-cdKO parasites were used to analyze cell cycle progression evaluated by DAPI staining during DXR treatment and after withdrawal. Parasites were classified according to the number of nuclei (N) and kinetoplasts (K): 1N1K (G1 phase); 1N1K* (asterisk denotes an enlarged K under segregation) and 1N2K (S phase); and 2N2K (G2/M phase). (B) dGTP and dTTP levels were monitored upon DXR exposition and after 16 h of agent removal in Tb BF, Tb BF HD82-dKO as well as Tb BF HD82-cdKO ± DOX parasites. Data are presented as the mean percentage (± SD) of total cells counted from three independent experiments (n > 150 cells in total). The asterisks show significant differences vs the parental line, calculated by the Student’s t test. *p < 0.05, **p < 0.01, ***p < 0.001. [file Image_6.tif]

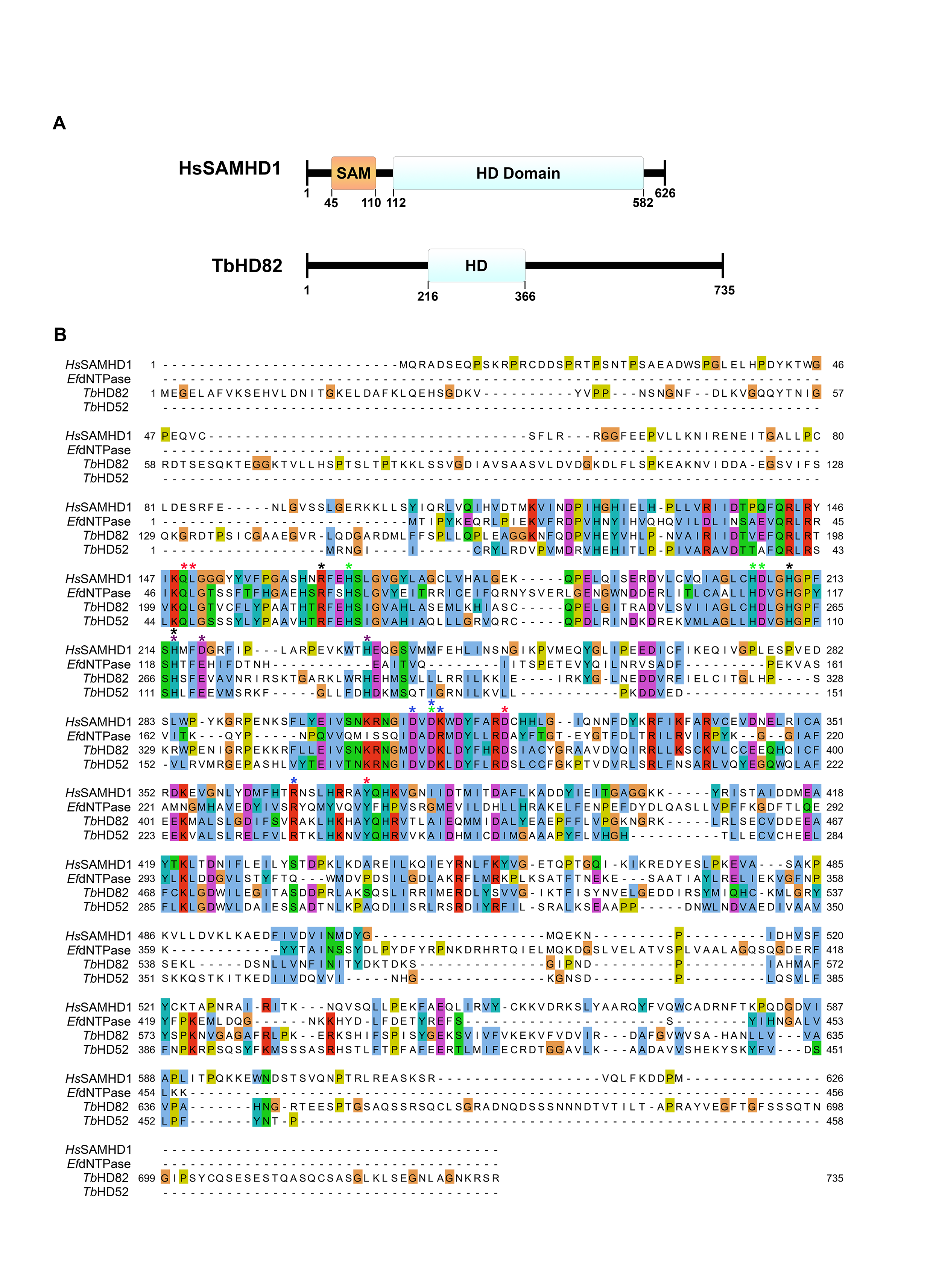

Supplement: Supplementary Figure 7 — (A) Comparison of protein length and the location of the HD domain between human SAMHD1 and TbHD82, with relevant amino acid positions marked. Prediction of the conserved domains was obtained with Pfam. (B) Alignment of TbHD82 with the representative HD containing proteins HsSAMHD1 and the dNTPase from Enterococcus faecalis. Alignment was carried out using ClustalOmega and displayed with JalView. Sequences were collected from UniProt database with the following protein IDs: HsSAMHD1 (Q9Y3Z3), EfdNTPase (Q836G9), TbHD82 (Q583P4) and TbHD52 (Q57X97). Asterisks indicate amino acids involved in catalysis and substrate binding: in red, residues involved in ribose exclusion; in black, residues involved in α-phosphate binding; in green, HD residues; in purple, catalytic residues; in blue, residues involved in Mg2+ coordination with the β and γ-phosphate groups. [file Image_7.tif]
